# Supplementary material for: Hierarchical control of enzymatic actuators using DNA-based switchable memories
Source: Nat Commun. 2017 Oct 24;8:1117. doi: 10.1038/s41467-017-01127-w (PMC5714950; doi:10.1038/s41467-017-01127-w)
Supplement: Supplementary file 1 — Supplementary Information [file 41467_2017_1127_MOESM1_ESM.pdf]

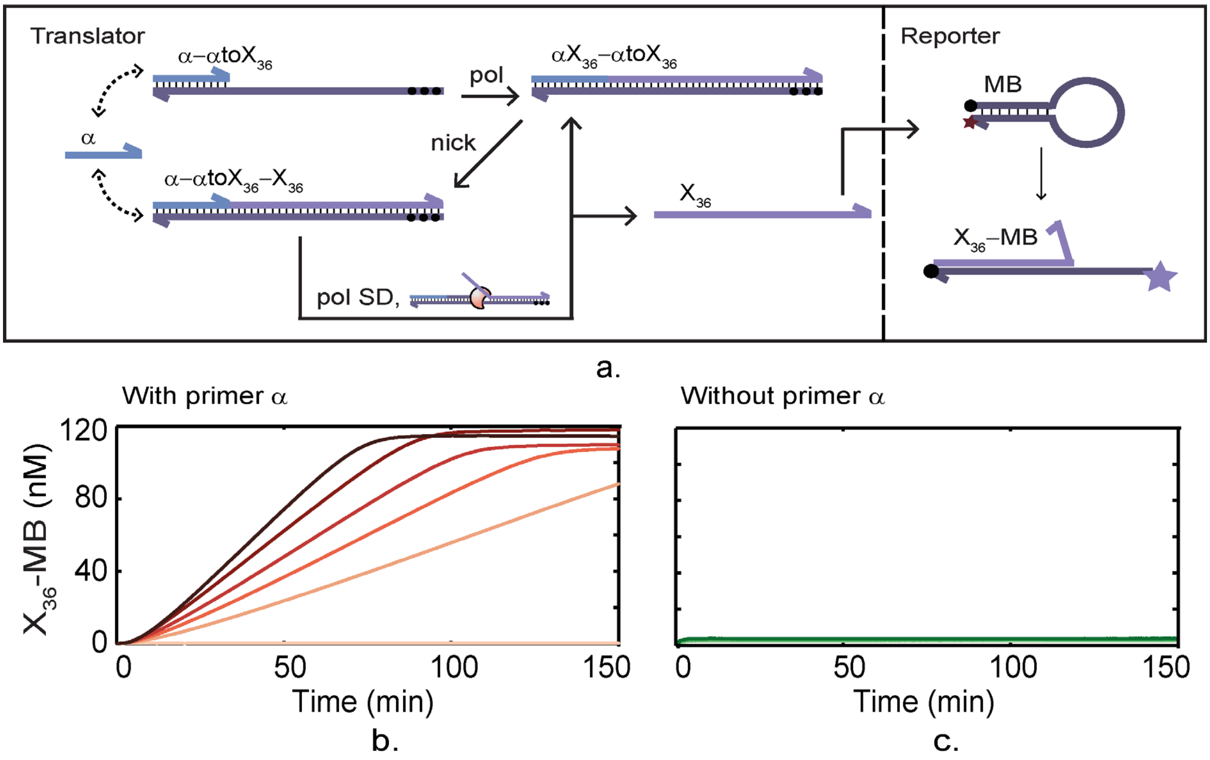

Supplementary Figure 1. Production of DNA strand X_{36} requires the presence of translator module and primer α. a, Schematic illustration of the reactions, which were carried out as described in the Methods (PEN-based experiments). The black dots at the 5'-end of the DNA strands represent phosphorothioate modifications which protect the DNA strand from degradation. An experiment was performed in the presence (**b**) and absence (**c**) of 50 nM of primer ***α*** and using 0, 5, 7.5, 10, 15 and 20 nM (from light to dark color) of translator template ***αtoX₃₆*** in the presence of molecular beacon ***MBₓ***, 10 U/mL Nt. bstNBI and 15 U/mL Bst. 2.0 warmstart DNA polymerase. Fluorescence was converted to concentration using a standard curve (Supplementary Fig. 17). The results in Figure S1 show an increase in fluorescence when both primer ***α*** and translator are present evidencing that the increase in fluorescence is only caused by the production of DNA strand ***X₃₆*** and, importantly, not by any unwanted background reaction.

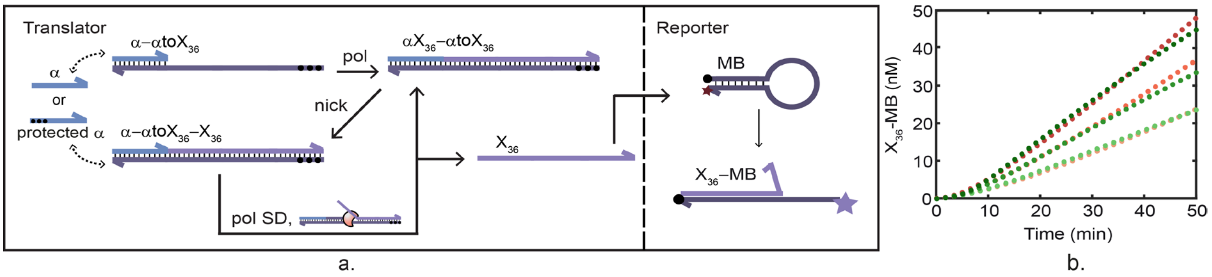

Supplementary Figure 2. Comparing the production rate of DNA strand X_{36} using primer α or protected primer α. Schematic illustration of the experiment in which the translator module produces DNA strand $\boldsymbol{X_{36}}$ using primer $\boldsymbol{\alpha}$ or protected primer $\boldsymbol{\alpha}$ and measured with a molecular beacon. Experiments were performed as described in the Methods (PEN-based experiments). The black dots represent phosphorothioate modifications. The experiment was performed using 5, 7.5 and 10 nM (from light to dark color) of translator template $\boldsymbol{\alpha to X_{36}}$ in the presence of 10 U/mL Nt.BstNBI, 15 U/mL Bst 2.0 warmstart and molecular beacon $\boldsymbol{MB_x}$ and initiated with 50 nM of unprotected primer $\boldsymbol{\alpha}$ or 50 nM of protected primer $\boldsymbol{\alpha}$. **b,** Results of the experiments which show a similar production rate of $\boldsymbol{X_{36}}$ using either unprotected (red dotted lines) or protected (green dotted lines) primer $\boldsymbol{\alpha}$.

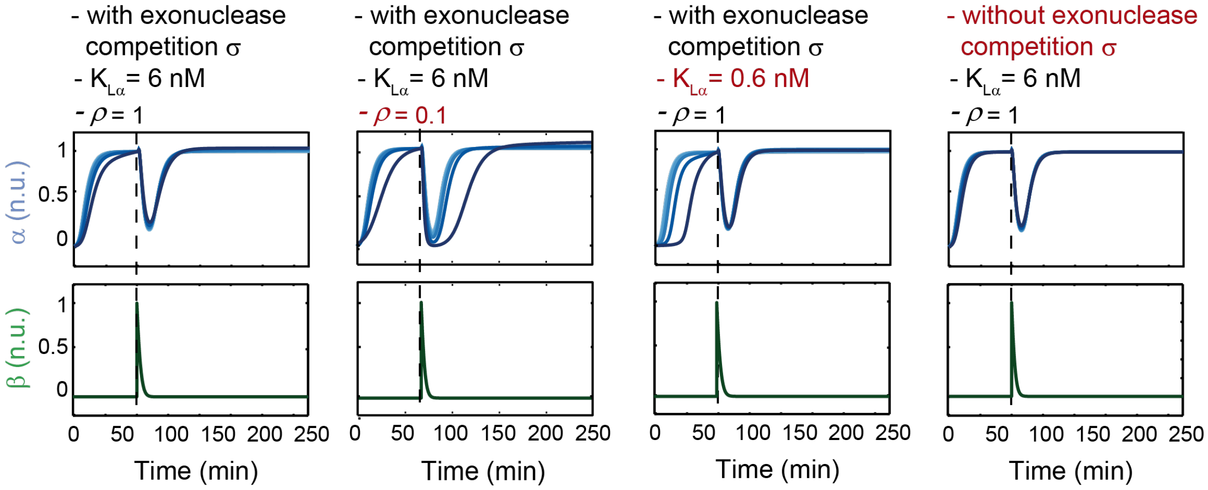

Supplementary Figure 3. Analysis of parameters potentially contributing to retroactivity. Simulations were performed using the heuristic model (Supplementary Notes) with the same concentrations of translator template as used for the experiments in Figure 3b. Besides the translator concentration the parameters ρ, $K_{L\alpha}$ and the competition term of $\boldsymbol{\sigma}$ in the Michaelis-Menten derivation of exonuclease can contribute to retroactivity in theory. The results of the simulations show the dynamics of the INVERTER circuit for a concentration range of translator module with one of these parameters modified as indicated in red. The traces were converted to normalized units (n.u.) by normalizing $\boldsymbol{\alpha}$ to the steady-state concentration and normalizing $\boldsymbol{\beta}$ to its maximum value. These results show that parameters ρ and $K_{L\alpha}$ have a significant contribution to retroactivity while the competition term of $\boldsymbol{\sigma}$ of the Michaelis-Menten derivation of exonuclease has a relatively small contribution to retroactivity.

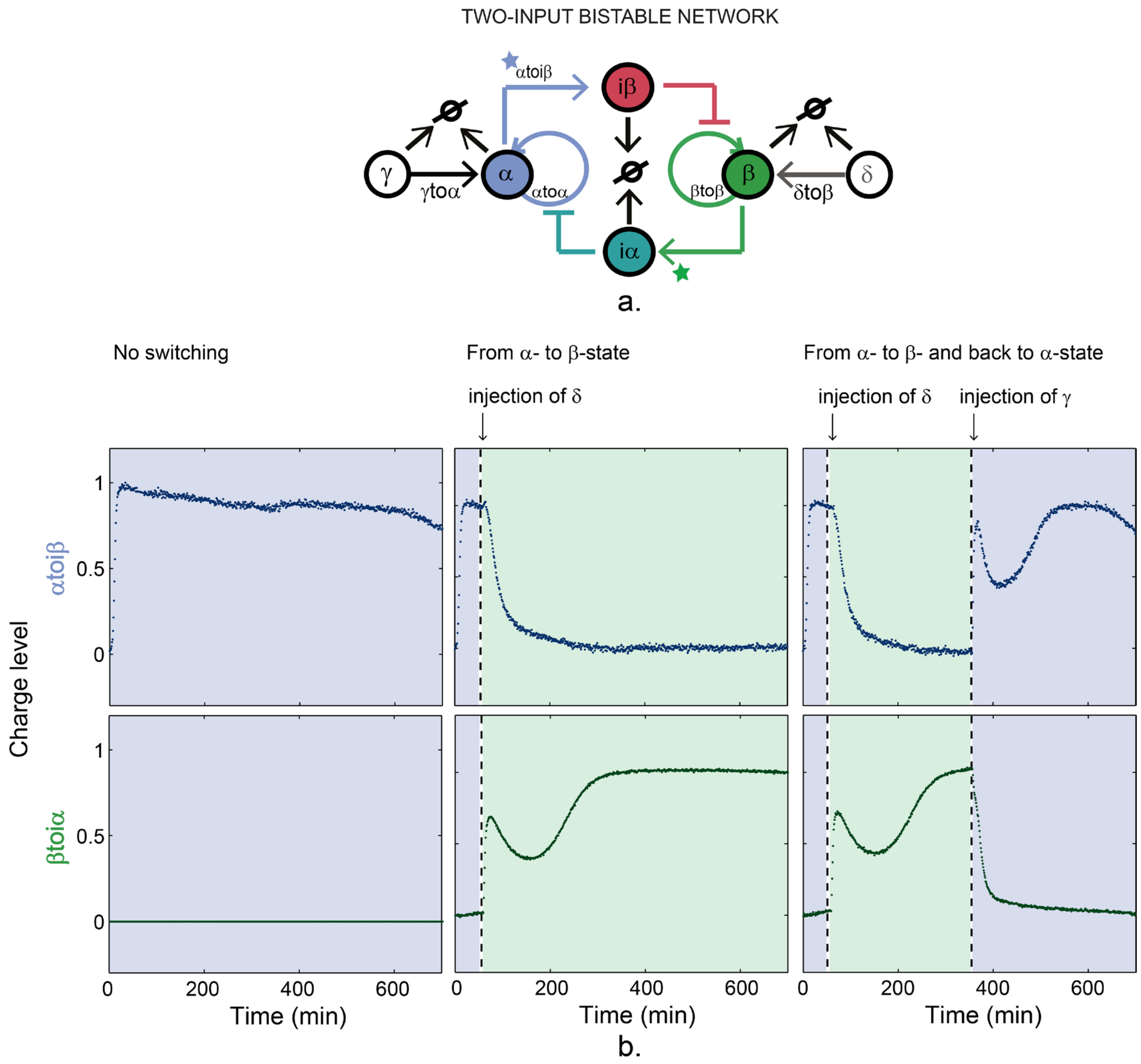

Supplementary Figure 4. The dynamics of the bistable switch for optimized template concentrations. a, Schematic illustration of the bistable switch which consists of four templates including the autocatalytic templates ***αtoα*** and ***βtoβ*** and the inhibitory templates ***αtoiβ*** and ***βtoiα*** from which inhibitors are produced cross-sequestering the autocatalytic templates. The network is defined to be in the α-state when ***αtoα*** is active and repressing ***βtoβ*** via ***αtoiβ***. Likewise, the network is defined to be in the β-state when ***βtoβ*** is active and repressing ***αtoα*** via ***βtoiα***. Furthermore, two more templates ***γtoα*** and ***δtoβ*** are included which serve as receivers for external input ssDNA ***γ*** and ***δ*** resulting in a long-lasting pulse of ***α*** or ***β*** unbalancing the circuit and stimulating the network to switch. **b**, Experiments were performed as described in the Methods (PEN-based experiments) and initiated with 1 nM of ***α***. To analyze the steady-states of the bistable switch the network was not switched (left), switched once by injection of 30 nM ***δ*** (middle) and switched twice by injection of 30 nM ***δ*** followed by injection of 30 nM ***γ*** (right). Experiments were performed using 20 nM ***βtoiα***, 15 nM ***αtoiβ***, 24 nM ***βtoβ***, 10 nM ***αtoα***, ***γtoα*** and ***δtoβ***, 10 U/mL Bst 2.0 warmstart DNA polymerase, 10

U/mL Nt. bstNBI and 200 nM ttRecJ. The charge level is the normalized fluorescence of the signal of DY530 and FAM fluorophores which is 0 in the absence of template's input primer and 1 at the steady-state value of primer ***β*** and ***α*** respectively. The results confirm that the bistable switch with asymmetric amounts of autocatalytic templates and inhibitory templates can keep its steady-states for an extended time period and, therefore, is indeed bistable until accumulation of waste, limiting amount of dNTP's and enzyme activity increasingly dominate the dynamics of the network.

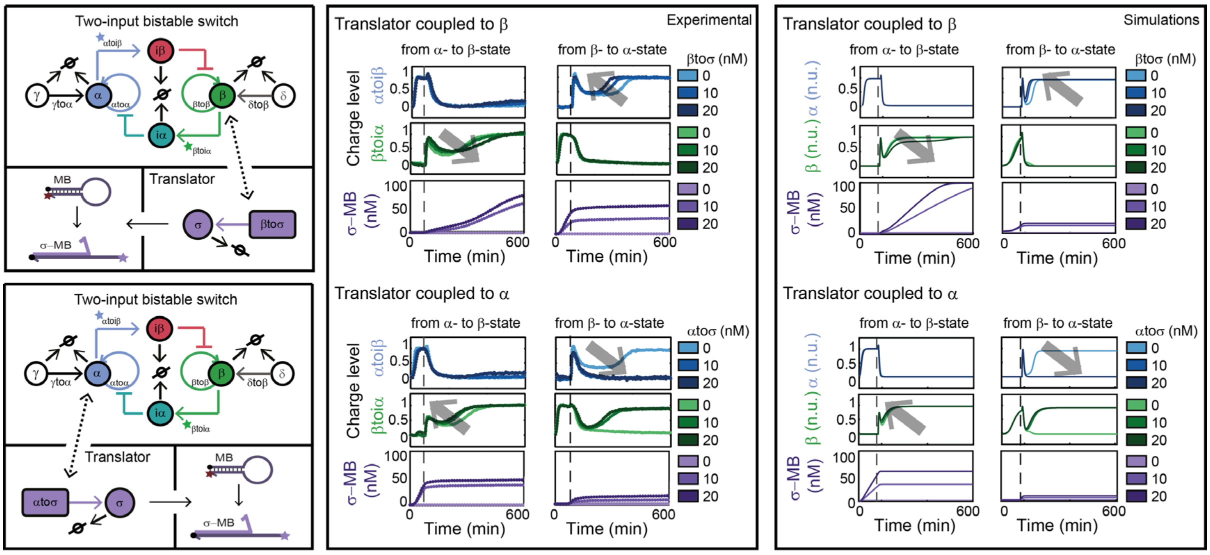

a. b. c.

Supplementary Figure 5. Coupling of the translator module to a two-input bistable switch. **a,** Schematic illustration of the system, in which the translator module is coupled to ***β*** or ***α*** of the PEN-based bistable switch. The core of the bistable switch consists of four templates including the autocatalytic templates ***αtoα*** and ***βtoβ*** and the inhibitory templates ***αtoiβ*** and ***βtoiα***. The network switches between states upon injection of ***γ*** and ***δ*** which are received by templates ***γtoα*** and ***δtoβ***. The dynamics of the bistable switch are followed via N-quenching using templates ***βtoiα*** and ***αtoiβ*** which are 3'-end labeled with a DY530 and FAM fluorophore respectively. **b,** Results of the experiments in which a concentration range of translator template was coupled to ***β*** or ***α***. The sequence of the translator template was adapted for coupling to ***β*** enabling the translator module to receive ***β*** as input primer while producing ***σ*** as output strand. Experiments were carried out as described in the Methods (PEN-based experiments) using 20 nM ***βtoiα***, 15 nM ***αtoiβ***, 24 nM ***βtoβ***, 10 nM ***αtoα***, ***γtoα*** and ***δtoβ***, 10 U/mL Bst 2.0 warmstart DNA polymerase, 10 U/mL Nt. bstNBI and 200 nM ttRecJ. The switch was initiated with 1 nM of ***α*** when the network was switched from the α- to the β-state, while the network was initiated with 1 nM of ***β*** when switched from the β- to the α-state. The dotted lines indicate the time point at which 30 nM ***δ*** (from α- to β-state) and ***γ*** (from β-

to α-state) was injected. The charge level is the normalized fluorescence of the signal of DY530 and FAM fluorophores which is 0 in the absence of template's input primer and 1 at the steady-state value of primer ***β*** and ***α*** respectively. The results of the translator coupled to ***α*** or ***β*** show the production rate of ***σ*** follows the dynamics of the switch instantaneously. Furthermore, the effect of retroactivity from the translator coupled to ***α*** or ***β*** was analyzed by plotting the data in the phase plane of ***αtoiβ*** and ***βtoiα*** (Fig. 4). **c**, Results of simulations performed using the heuristic model (Supplementary Notes). The traces of ***α*** and ***β*** were converted to normalized units (n.u.) by normalizing ***α*** and ***β*** to their steady-state concentration. The results show the same trend in the dynamics of the switch with increasing translator module as for the experiments.

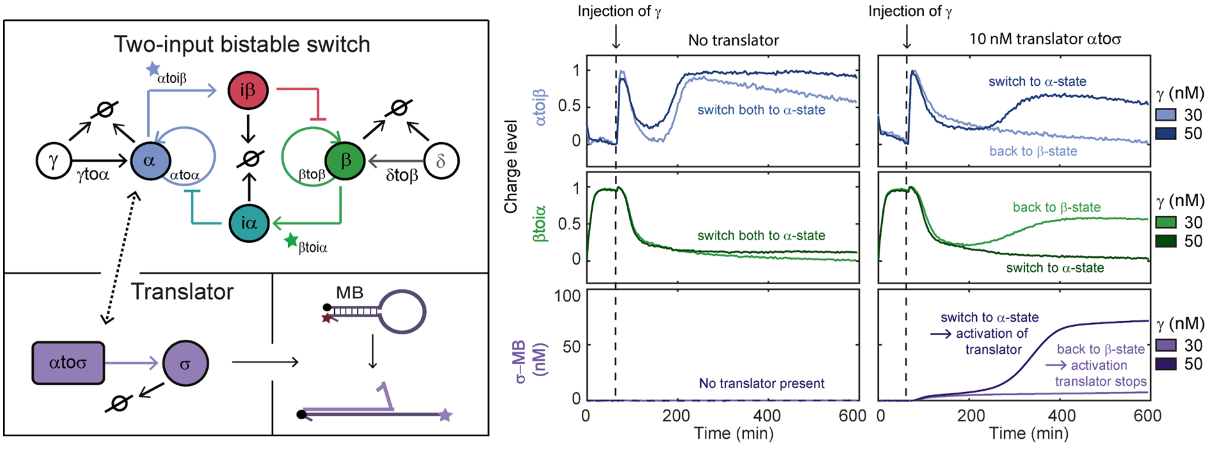

a. b.

Supplementary Figure 6. Switching from the β- to the α-state with no translator and with the translator module coupled to *α*. a, Schematic illustration of the system, in which the translator module is coupled to ***α*** of the PEN-based bistable switch. The core of the bistable switch consists of four templates including the autocatalytic templates ***αtoα*** and ***βtoβ*** and the inhibitory templates ***αtoiβ*** and ***βtoiα***. The network starts in the β-state and switches from the β- to the α-stat upon injection of ***γ*** which is received by template ***γtoα***. The dynamics of the bistable switch are followed via N-quenching using templates ***βtoiα*** and ***αtoiβ*** which are 3'-end labeled with a DY530 and FAM fluorophore respectively. **b,** Results of the experiments in which switching from the β- to the α-state was initiated with 30 nM or 50 nM ***γ*** in absence of translator module and with 10 nM of the translator module (***αtoσ***) coupled to ***α***. Experiments were carried out as described in the Methods (PEN-based experiments) using 20 nM ***βtoiα***, 15 nM ***αtoiβ***, 24 nM ***βtoβ***, 10 nM ***αtoα***, ***γtoα*** and ***δtoβ***, 10 U/mL Bst 2.0 warmstart DNA polymerase, 10 U/mL Nt. bstNBI and 200 nM ttRecJ. The switch was initiated with 1 nM of ***β***. The dotted lines indicate the time point at which 30 nM or 50 nM ***γ*** was injected. The charge level is the normalized fluorescence of the signal of DY530 and FAM fluorophores which is 0 in the absence of template's input primer and 1 at the steady-state value of primer ***β*** and ***α*** respectively. The results show switching from the β- to the α-state without translator module after injection of both 30 nM and 50 nM of ***γ***, while switching from the β- to the α-state with 10 nM of the translator module coupled to ***α*** only succeeded after injection of 50 nM of ***γ***. Hence, these results verify the predictions of the theoretical model as illustrated in the bifurcation diagrams in Figure 4.

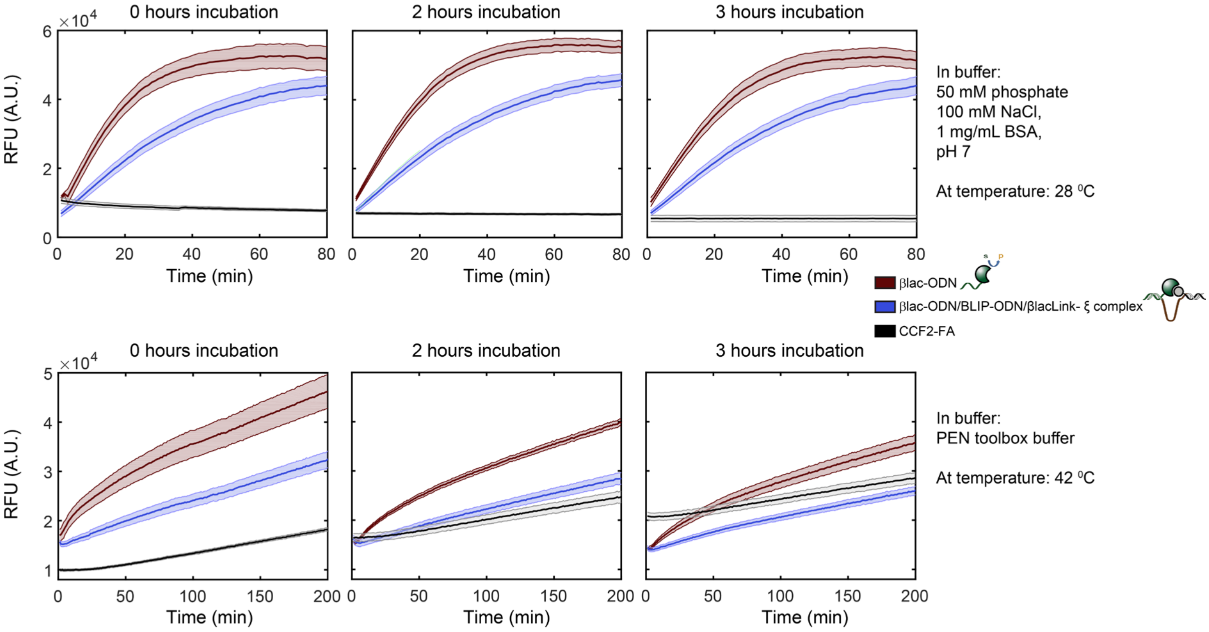

Supplementary Figure 7. Comparing the β-lactamase actuator in the original buffer at 28 °C and in the PEN toolbox buffer at 42 °C. 1 nM of β-lactamase-ODN (dark red) or 1 nM β-lactamase-ODN in complex with BLIP-ODN and DNA linker (βlacLink-ξ) in ratio 1:2:1.2 were incubated in a 396 wells-plate with a volume of 36 µL and 25 µL oil to prevent evaporation for different time periods as shown above the graphs. The experiment was performed in either the original buffer[3] (upper graphs) or PEN toolbox buffer (lower graphs, Methods (PEN-based experiments)). Enzymatic activity of TEM1 β-lactamase was measured by adding 4 µL fluorescent substrate CCF2-FA (final concentration of 2 µM) prior to the measurement (Tecan, Safire). Furthermore, experiments were performed where CCF2-FA substrate was incubated on its own as shown by the grey traces. Shaded area's represent the standard deviation of the mean of the experiments. The results show that the β-lactamase-ODN (dark red) and the β-lac/BLIP/template complex (blue) are relatively stable in the original buffer at 28 °C for at least 3 hours. However, β-lactamase-ODN (dark red) and the self-inhibitory β-lactamase construct (blue) show a decrease in activity in PEN toolbox buffer at 42 °C over time. Furthermore, while CCF2-FA is stable in the original buffer at 28 °C it is slowly hydrolyzed in PEN toolbox buffer at 42 °C.

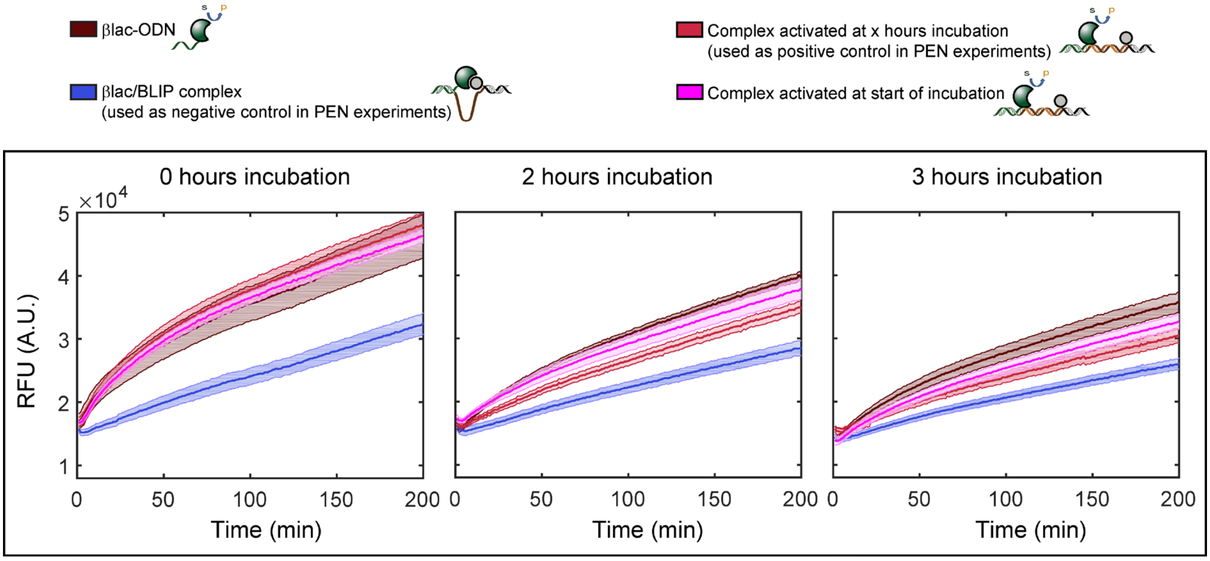

Supplementary Figure 8. The activation of the β-lactamase actuator in the PEN toolbox buffer at 42 °C for different incubation periods. 1 nM of β-lactamase-ODN or 1 nM β-lactamase-ODN in complex with BLIP-ODN and DNA linker ***βlacLink-ξ*** (ratio 1:2:1.2) were incubated in a volume of 36 µL with 25 µL oil to prevent evaporation for different time periods as shown above the graphs. The experiment was performed in the PEN toolbox buffer (Methods). Samples included the β-lactamase-ODN (dark red), the inactivated complex (blue), the complex activated (with 50 nM ***ξ***) at the start of the incubation (pink) and the complex activated (with 50 nM ***ξ***) at 'X' hours (red) as indicated above the graphs. Enzymatic activity of TEM1 β-lactamase was measured by adding 4 µL fluorescent substrate CCF2-FA (final concentration of 2 µM) prior to the measurement (Tecan, Safire). Shaded area's represent the standard deviation of the mean of the experiments. The results show that TEM1 β-lactamase decreases in activity over incubation time. However, the activated complexes (pink and red traces) after 2 and 3 hours incubation do not reach the activity of the free β-lactamase-ODN. Moreover, the increase in activity by activation of the complex becomes lower with increasing incubation time of the inactivated complex (red traces). Possibly the TEM1 β-lactamase is less stable in complex with BLIP compared to the free TEM1 β-lactamase-ODN. Furthermore, the complex possibly dissociates over time explaining why the negative control (blue) decreases less in activity compared to the free TEM1 β-lactamase-ODN (dark red). For these reasons, as a positive control we use the complex activated (by ***ξ***) after 'x' hours in the PEN toolbox environment.

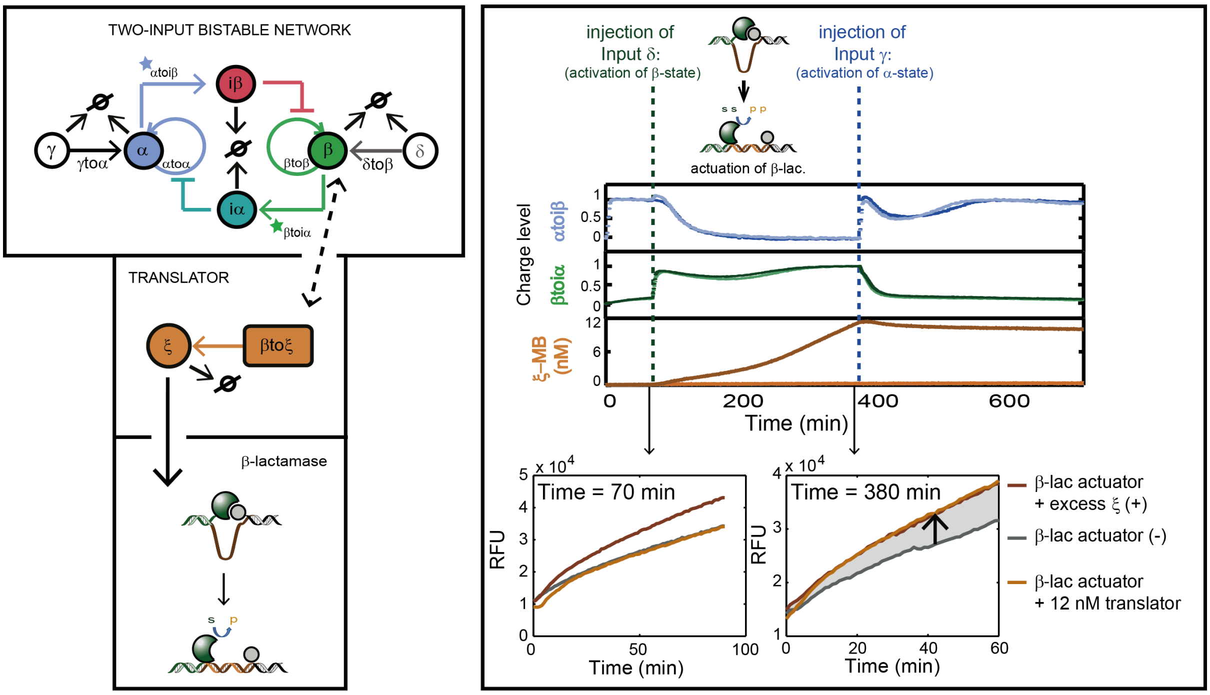

a. b.

Supplementary Figure 9. Controlling the activity of a TEM1 β-lactamase enzyme by a two-input bistable switch. a, Schematics of the experiment in which the two-input bistable network is used to control the activity of a self-inhibitory TEM1 β-lactamase construct. **b,** Results of the experiments which were carried out as described in the Methods (PEN-based experiments) using 20 nM ***βtoiα***, ***αtoiβ***, and ***βtoβ***, 10 nM ***αtoα***, ***γtoα*** and ***δtoβ***, 15 U/mL Bst 2.0 warmstart DNA polymerase, 10 U/mL Nt. bstNBI, 75 nM ttRecJ, 5 nM self-inhibitory TEM1 β-lactamase construct or 20 nM molecular beacon $\boldsymbol{MB_\xi}$ in absence and presence of 10 nM ***βtoξ*** and initiated with 1 nM of ***α***. The upper three graphs show the dynamics of the bistable switch and the production of DNA strand ***ξ*** measured using a molecular beacon labeled with ROX fluorophore and a quencher. The dotted lines show the time at which the Inputs ***δ*** and ***γ*** were added. The charge level is the normalized fluorescence of the signal of DY530 and FAM fluorophores which is 0 in the absence of template's input primer and 1 at the steady-state value of primer ***β*** and ***α*** respectively. The fluorescence of the ROX fluorophore was converted to concentration of DNA strand ***ξ*** using a standard curve (Supplementary Fig. 17). The experiments were performed in presence (dark blue, green and dark yellow) and absence (light blue, light green and light yellow) of translator module and the results show that the dynamics of the bistable switch are not disturbed by addition of the translator module. In parallel, experiments were run were the molecular beacon was replaced with the self-inhibitory TEM1 β-lactamase construct. The activity of the TEM1 β-lactamase was determined at two different states of the switch including 70 and 380 minutes after initiation of the switch, indicated by the black arrows, by measuring the conversion rate of fluorogenic substrate CCF2-FA. Negative and positive controls were carried out in

parallel. For negative controls (grey) the translator module was omitted in the reactions. For positive controls (red) the translator module was also omitted and excess of DNA strand ξ (100 nM) was added to the wells plate. The results show we are able to control the activity of the self-inhibitory TEM1 β-lactamase construct by the dynamics of the bistable switch.

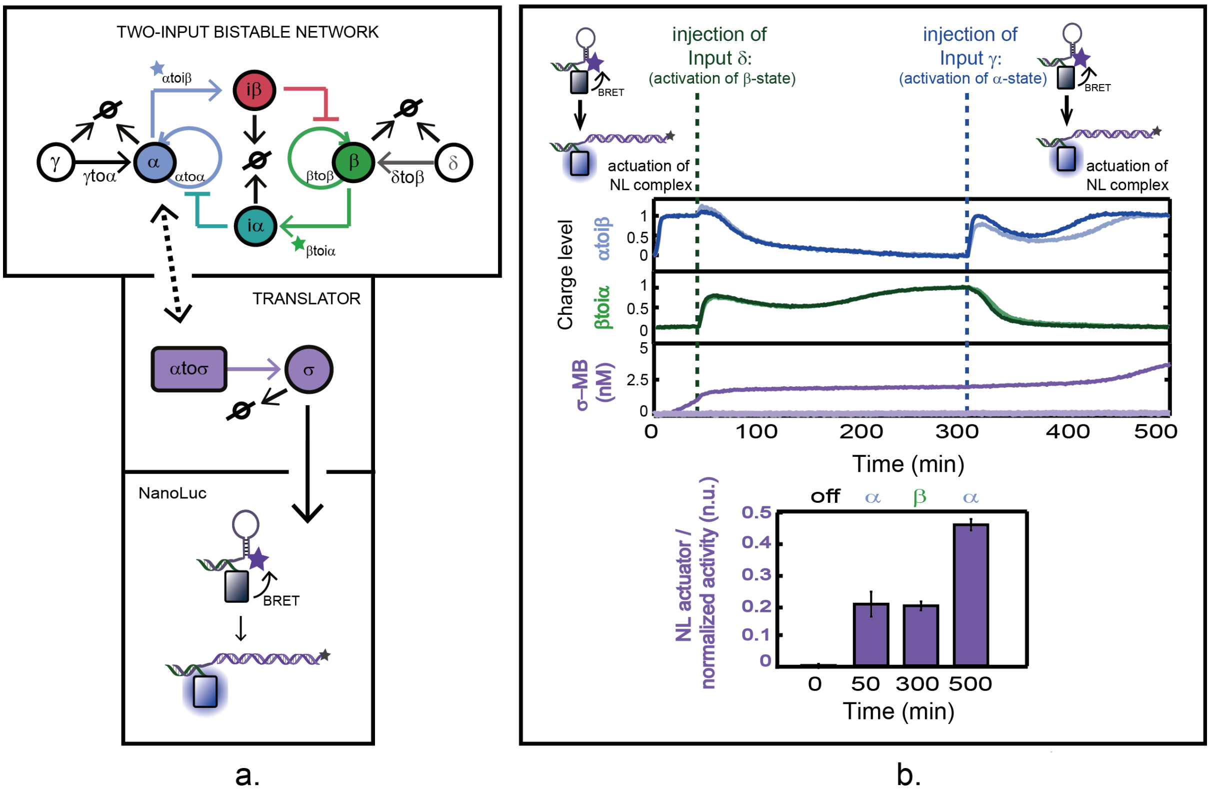

Supplementary Figure 10. Control of a NanoLuc-based enzymatic actuator by a two-input bistable switch. a, Schematics of the experiment in which the two-input bistable network is used to control a NanoLuc-based enzymatic actuator. **b,** Results of the experiments which were carried out as described in the Methods using 20 nM ***βtoiα***, 15 nM ***αtoiβ***, 24 nM ***βtoβ***, 10 nM ***αtoα***, ***γtoα*** and ***δtoβ***, 15 U/mL Bst 2.0 warmstart DNA polymerase, 10 U/mL Nt. bstNBI, 200 nM ttRecJ, 5 nM NanoLuc actuator or molecular beacon MB_{σ} in absence and presence of 2 nM ***αtoσ*** and initiated with 1 nM of ***α***. The upper three graphs show the dynamics of the bistable switch and the production of DNA strand ***σ*** measured using a molecular beacon labeled with Cy5 fluorophore and a quencher. The dotted lines show the time at which the Inputs ***δ*** and ***γ*** were added. The charge level is the normalized fluorescence of the signal of DY530 and FAM fluorophores which is 0 in the absence of template's input primer and 1 at the steady-state value of primer ***β*** and ***α*** respectively. The fluorescence of the Cy5 fluorophore was converted to concentration of DNA strand ***σ*** using a standard curve (Supplementary Fig. 17). The experiments were performed in presence (dark blue, green and yellow) and absence (light blue, light green and light yellow) of translator module and the

results show that the dynamics of the bistable switch are not disturbed by addition of the translator module. In parallel, experiments were run were the molecular beacon was replaced with the NanoLuc-based enzymatic actuator. The conformational state of the actuator was determined at different states of the switch including 0, 50, 300 and 500 minutes after initiation of the switch by primer ***α***, shown in the bar graph. The fraction of opened conformation in normalized units (n.u.) was calculated by subtracting the mean BRET ratio of the positive controls and normalizing to the mean BRET ratio of the negative controls (Supplementary Fig. 11). For negative and positive controls the translator module was omitted and excess of DNA strand ***σ*** was added for the positive control. Error bars represent the standard deviation of the mean of the experiments performed in triplicate. The results show we are able to control the NanoLuc-based actuator by the dynamics of the bistable switch.

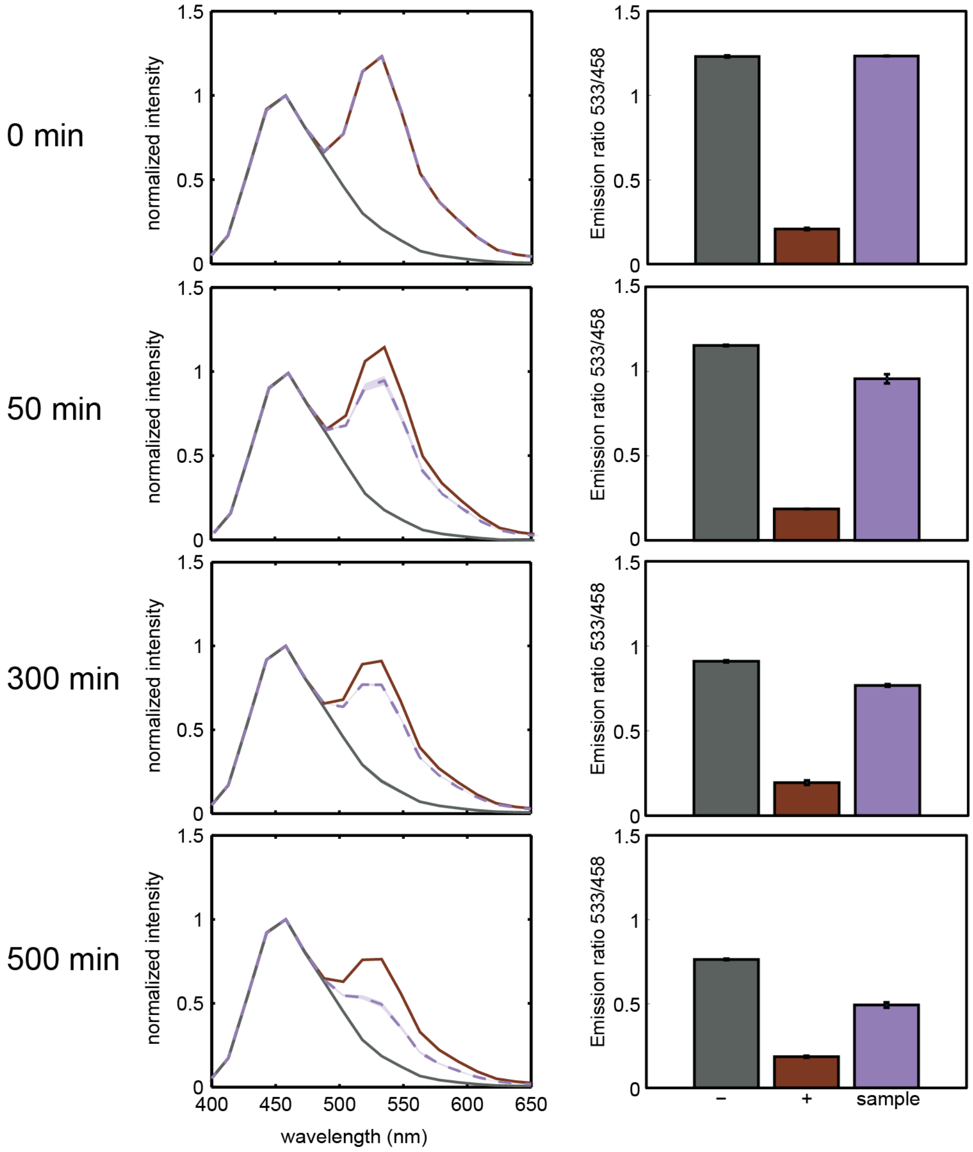

Supplementary Figure 11. Normalized raw data of the control of the NanoLuc-complex at different states of the switch (Fig S10). A detailed description of the protocol can be found in the Methods. For negative (–, grey) and positive (+, red) controls the translator module was omitted and excess of DNA strand $\boldsymbol{\sigma}$ was added to the wells plate for the positive controls. The BRET ratio at 533 nm and 458 nm was calculated for samples and controls. Experiments were performed in triplicate. Error bars and shaded area's represent the standard deviation of the mean of the experiments.

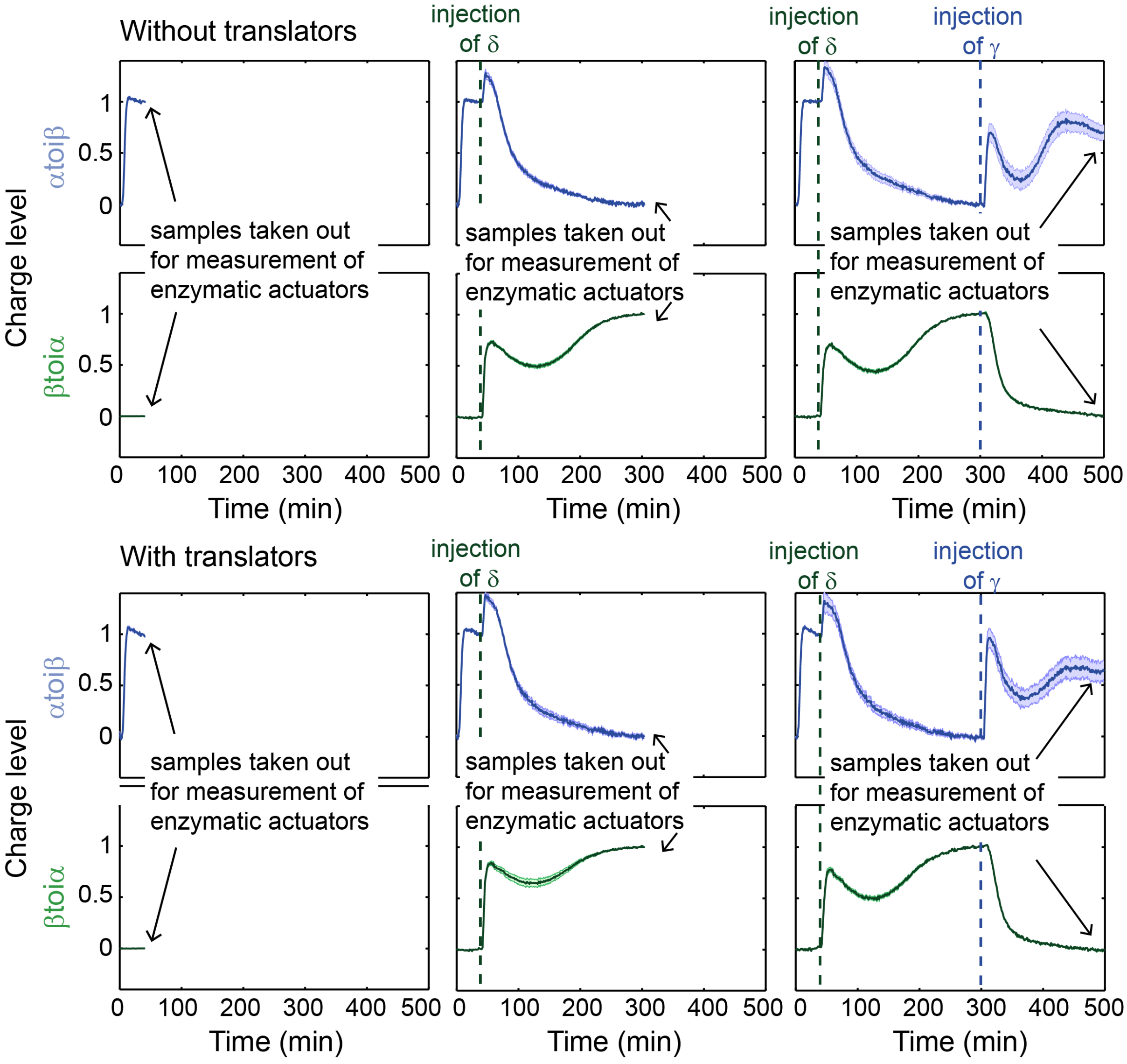

Supplementary Figure 12. Raw data displaying the dynamics of the switch while probing the activity of the enzymatic actuators (Fig. 6). Experiments were carried out as described in the Methods in absence of translator templates (for the measurement of the positive and negative controls in determining the activity of the enzymatic actuators) and in presence of the orthogonal set of translator templates including ***αtoσ*** and ***βtoξ***. The charge level is the normalized fluorescence of the signal of DY530 and FAM fluorophores which is 0 in the absence of template's input primer and 1 at the steady-state value of primer ***β*** and ***α*** respectively. The dotted lines show the time points at which 30 nM of Input ***δ*** or ***γ*** was added. Shaded area's represent the standard deviation of the mean of the experiments. Experiments were performed in duplicate at three different days.

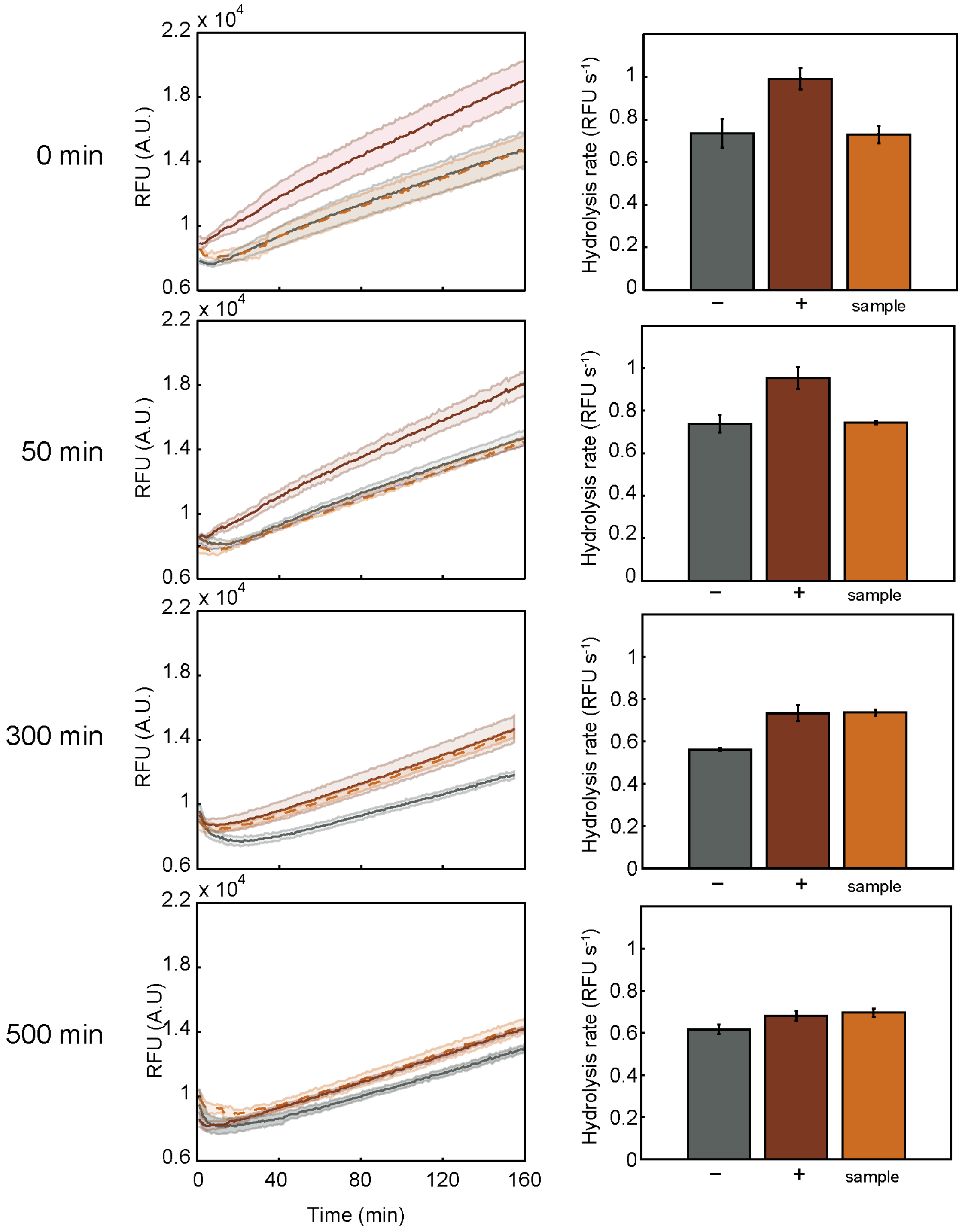

Supplementary Figure 13. Raw data of the activity of β-lactamase at different states of the switch (Fig. 6). The activity of β-lactamase was measured by adding CCF2-FA to the samples after which fluorescence at 447 nm was measured. A detailed description of the protocol can be found in the Methods. Conversion of CCF2-FA is shown on the left for the different states of the switch. For negative (–, grey) and positive (+, red) controls the translator templates ***αtoσ*** and ***βtoξ*** were omitted and excess of DNA strand ***ξ*** was added to the wells plate for the positive controls. The activity of β-lactamase was determined by deriving the slope between 50 and 150 minutes, shown on the right.

Experiments were performed in duplicate at three different days. Error bars and shaded area's represent the standard deviation of the mean of the experiments.

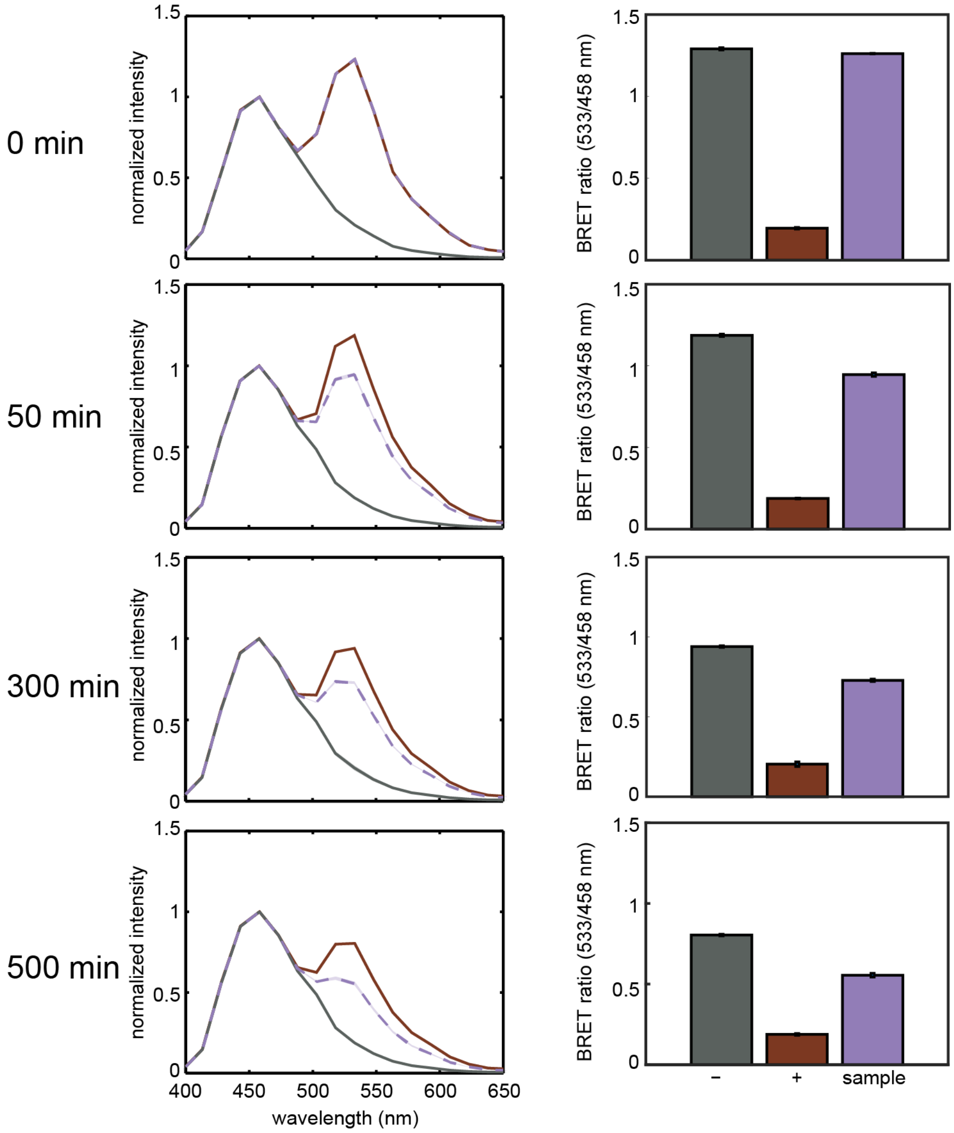

Supplementary Figure 14. Normalized raw data of the control of the NanoLuc-complex at different states of the switch (Fig. 6). A detailed description of the protocol can be found in the Methods. For negative (–, grey) and positive (+, red) controls the translator templates ***αtoσ*** and ***βtoξ*** were omitted and excess of DNA strand ***σ*** was added to the wells plate for the positive controls. The BRET ratio at 533 nm and 458 nm was calculated for samples and controls. Experiments were performed in duplicate at three different days. Error bars and shaded area's represent the standard deviation of the mean of the experiments.

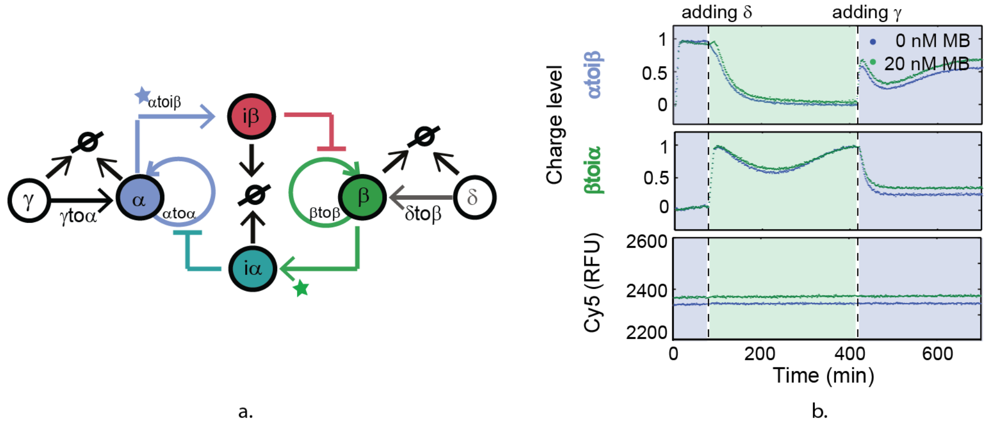

Supplementary Figure 15. Characterizing the crosstalk between the molecular beacon and the switch. a, Schematic illustration of the switch. **b,** Results of the experiments which were performed as described in the Methods (PEN-based experiments) in absence of molecular beacon (blue) and in presence of 20 nM molecular beacon $\boldsymbol{MB_\sigma}$ (green). The black dotted lines are the timepoints at which primers ***δ*** and ***γ*** were added respectively. Experiments were performed using 20 nM of ***βtoiα***, ***αtoiβ*** and 20 nM ***βtoβ***, 12 nM ***αtoα***, 10 U/mL Bst 2.0 warmstart DNA polymerase, 10 U/mL Nt. bstNBI and 50 nM ttRecJ. The experiment is initiated with 1 nM of primer ***α*** and, hence, the ***α***-side of the switch is initiated first. The charge level is the normalized fluorescence of the signal of DY530 and FAM fluorophores which is 0 in the absence of template's input primer and 1 at the steady-state value of primer ***β*** and ***α*** respectively. The experiments show the molecular beacon does not interact with the switch and does not change the dynamics significantly. Furthermore, the results on the bottom show the molecular beacon is not opened in these reaction mixtures.

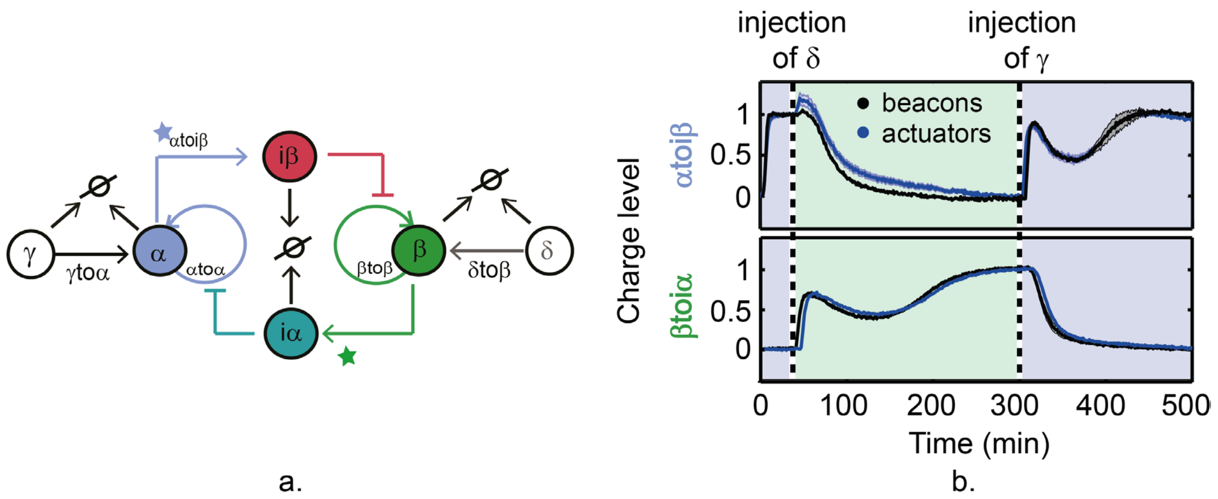

Supplementary Figure 16. Characterizing crosstalk between the enzymatic actuators and the switch. a, Schematic illustration of the switch. **b,** Results of the experiments which were performed as described in the Methods (PEN-based experiments) in presence of molecular beacons (black) or in presence of the enzymatic actuators (blue). The black dotted lines are the timepoints at which primers ***δ*** and ***γ*** were added respectively. Experiments were performed using 20 nM ***βtoiα***, 15 nM ***αtoiβ***, 24 nM ***βtoβ***, 10 nM ***αtoα***, ***γtoα*** and ***δtoβ***, 15 U/mL Bst 2.0 warmstart DNA polymerase, 10 U/mL Nt. bstNBI, 200 nM ttRecJ in presence of 5 nM NanoLuc actuator and 2.5 nM β-lactamase actuator or 5 nM MB_{σ} and 2.5 nM MB_{ξ}. The experiment is initiated with 1 nM of primer ***α*** and, hence, the α-side of the switch is initiated first. The charge level is the normalized fluorescence of the signal of DY530 and FAM fluorophores which is 0 in the absence of template's input primer and 1 at the steady-state value of primer ***β*** and ***α*** respectively. Experiments were performed in duplicate. Shaded area's represent the standard deviation of the mean of the experiments. The experiments show that both enzymatic actuators have low cross-talk with the switch as evidenced by similar switching dynamics in the presence of beacons and actuators.

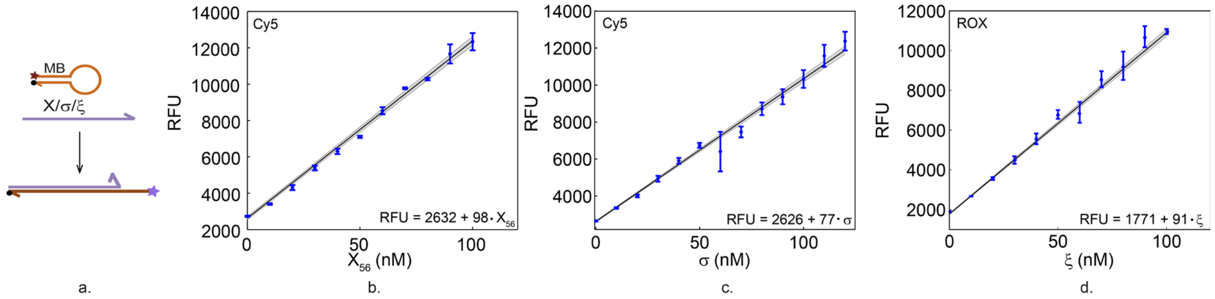

Supplementary Figure 17. Standard curve of molecular beacons with different amounts of targets. **a,** Experiments were performed in mastermix without enzymes (Methods) and fluorescence was measured at 42 °C. **b,** Standard curve of molecular beacon ***MB***$_{x}$ and target ***X***$_{56}$. **c,** Standard curve of molecular beacon ***MB***$_{\sigma}$ and target ***σ***. **d,** Standard curve of molecular beacon ***MB***$_{\xi}$ and target ***ξ***. **b-d,** To obtain the slope the experimental data was fitted to a linear equation.

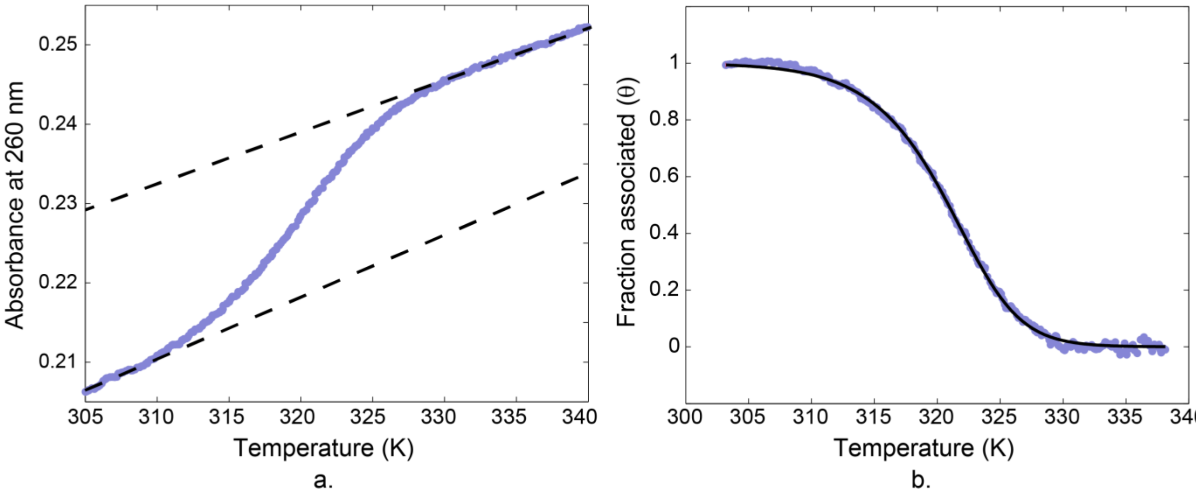

Supplementary Figure 18. Characterization of the thermodynamics of DNA hybridization. **a**, Thermal denaturation curve (blue) of the partially duplex ***α.γtoα*** in TE buffer complemented with 0.06 M NaCl and 0.008 M $MgSO_4$. The lower and upper baselines (black dotted), corresponding to associated and unassociated forms respectively, are used to determine the melting temperature (intersection of median of black dotted lines with experimental curve). **b**, The baselines were used to convert the absorbance to fraction associated, i.e. bimolecular duplex (blue), which was used for non-linear least squares fitting to obtain the standard Gibbs free energy (black) from which the thermodynamic dissociation constant was obtained (Supplementary Table 2). A detailed description of the experiment and analysis is provided in the Supplementary Notes.

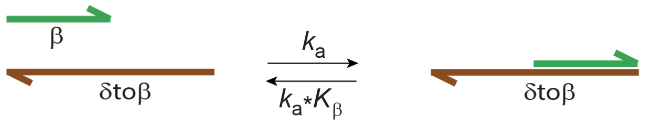

a.

$$\frac{d[\beta]}{dt} = -k_a[\beta][\delta to\beta] + k_a K_\beta[\delta to\beta]$$

$$\frac{d[\delta to\beta]}{dt} = -k_a[\beta][\delta to\beta] + k_a K_\beta[\delta to\beta \cdot \beta]$$

$$\frac{d[\delta to\beta \cdot \beta]}{dt} = k_a[\beta][\delta to\beta] - k_a K_\beta[\delta to\beta \cdot \beta]$$

b.

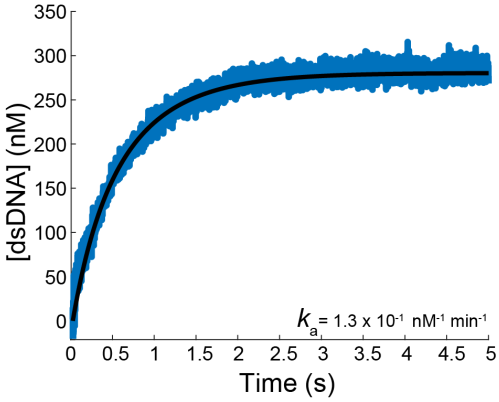

c.

Supplementary Figure 19. Characterization of the kinetics of DNA hybridization. **a,** Schematic illustration of DNA hybridization. **b,** Ordinary differential equations describing the kinetics of DNA hybridization. **c,** Results of non-linear least squares analysis (Supplementary Table 2) of the data to the ODE model based on the equations in (b). Experiments were performed using 500 nM primer *β* and template *δtoβ* in 1x TE buffer with 0.06 nM Na^+ and 0.008 M Mg^{2+}. A detailed description of the experiment and analysis is provided in the Supplementary Notes.

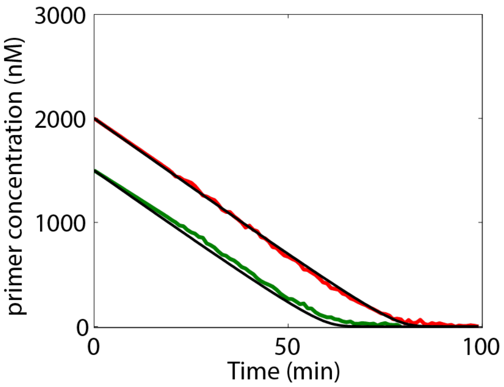

Supplementary Figure 20. Michaelis-Menten kinetics of ttRecJ exonuclease. To determine the enzymatic parameters of ttRecJ, the degradation of 1500, 2000 and 2500 nM (light to dark red) of DNA strand ***iβ*** was followed in time by the decrease in fluorescence from the dye Evagreen. The datacurves were used in the nonlinear least square optimization (black) to obtain an estimate of the Michaelis-Menten parameter K_M and V_{max} (Supplementary Table 2). A detailed description of the experiment and analysis is provided in the Supplementary Notes.

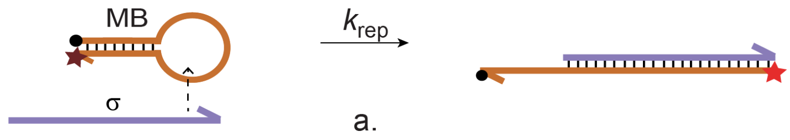

$$\frac{d[MB]}{dt} = -k_{rep}[\sigma][MB]; \quad \frac{d[\sigma]}{dt} = -k_{rep}[\sigma][MB]; \quad \frac{d[\sigma.MB]}{dt} = k_{rep}[\sigma][MB]$$

b.

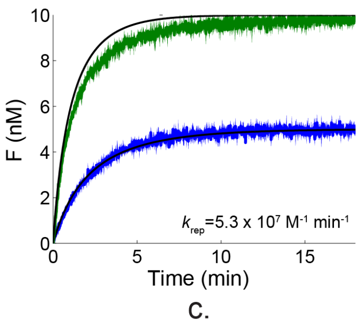

Supplementary Figure 21. Characterization of the kinetics of the molecular beacon. **a,** Schematic illustration of the experiment in which DNA strand ***σ*** binds to the loop of the beacon followed by strand displacement disrupting the stem of the beacon resulting in an increase in fluorescence. **b,** Ordinary differential equations describing the kinetics of the molecular beacon using the bimolecular reaction approximation.[1] **c,** Experiments were performed using 10 nM of molecular beacon and 5 (blue) and 20 nM (green) of DNA strand ***σ*** respectively in mastermix without enzymes and fluorescence was recorded over time. Non-linear least squares optimization was performed using the ODE model in order to obtain an estimate for the second-order rate k_{rep} (Supplementary Table 2). A detailed description of the experiment and analysis is provided in the Supplementary Notes.

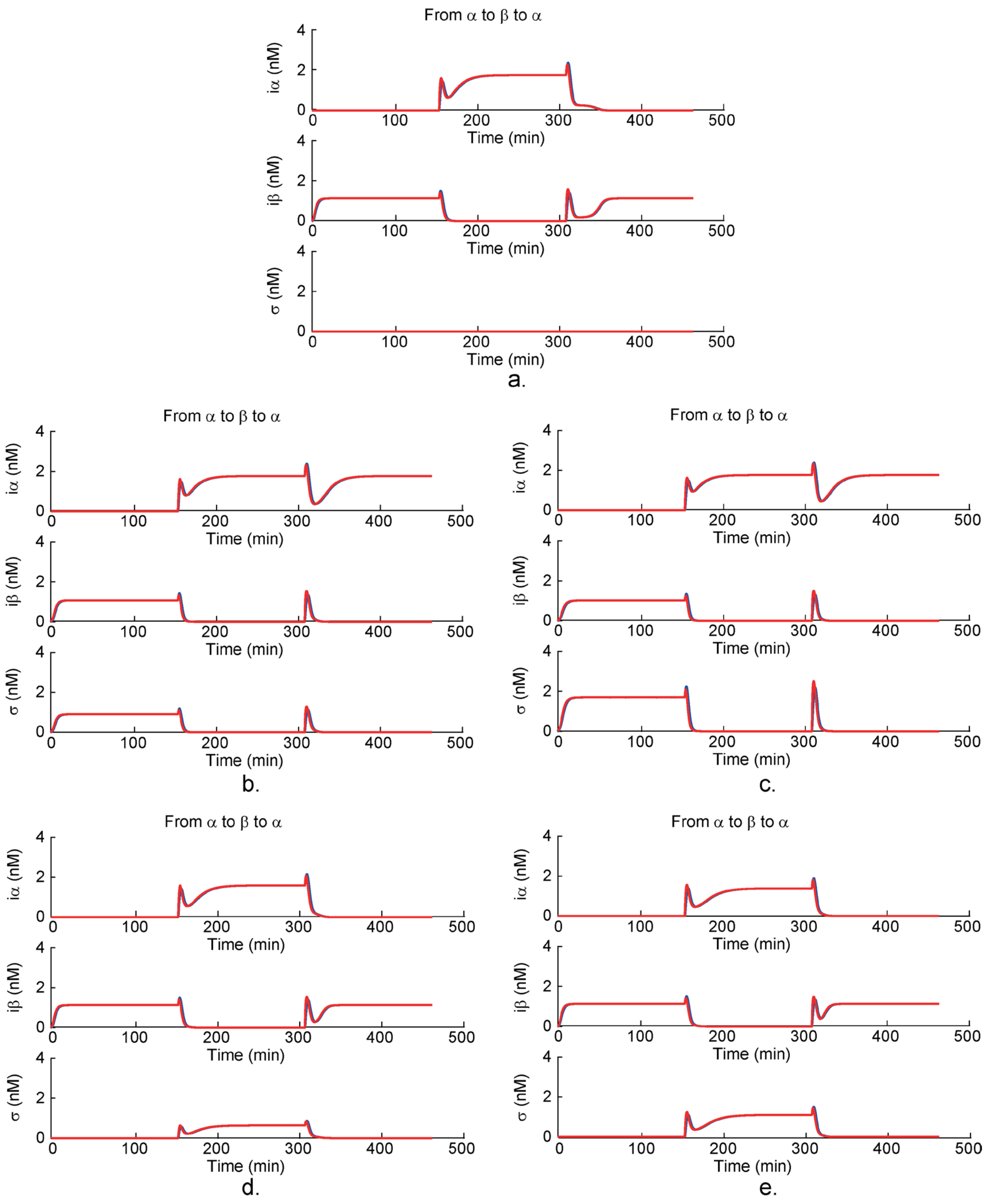

Supplementary Figure 22. Comparison of dynamics of full heuristic model and the reduced model. ***iα***, ***iβ*** and ***σ*** obtained with the full heuristic model (blue) and its steady-state approximations $\mathbf{f}_{i\alpha}$, $f_{i\beta}$, and f_{σ} (red) for switches from ***α*** to ***β*** to ***α***. **b.** 10 nM translator at ***α*** ($V_{L\alpha}$ = 0.65 nM min^{-1}). **c.** 20 nM translator at ***α*** ($V_{L\alpha}$ = 1.3 nM min^{-1}). **d.** 10 nM translator at ***β*** ($V_{L\beta}$ = 0.65 nM min^{-1}). **e.** 20 nM translator at ***β*** ($V_{L\beta}$ = 1.3 nM min^{-1}). Switches are initiated at t = 160 min and t = 310 min. Switching from ***α*** to ***β*** is initiated by a ***δ***-pulse and switching from ***β*** to ***α*** is initiated by a ***γ***-pulse. The dynamical behavior of the heuristic model and its reduction are qualitative similar. Using the reduced model separatrices and switching planes are obtained (Supplementary Fig. 26-29). A detailed description of the heuristic model and its steady-state approximations is provided in Supplementary Notes.

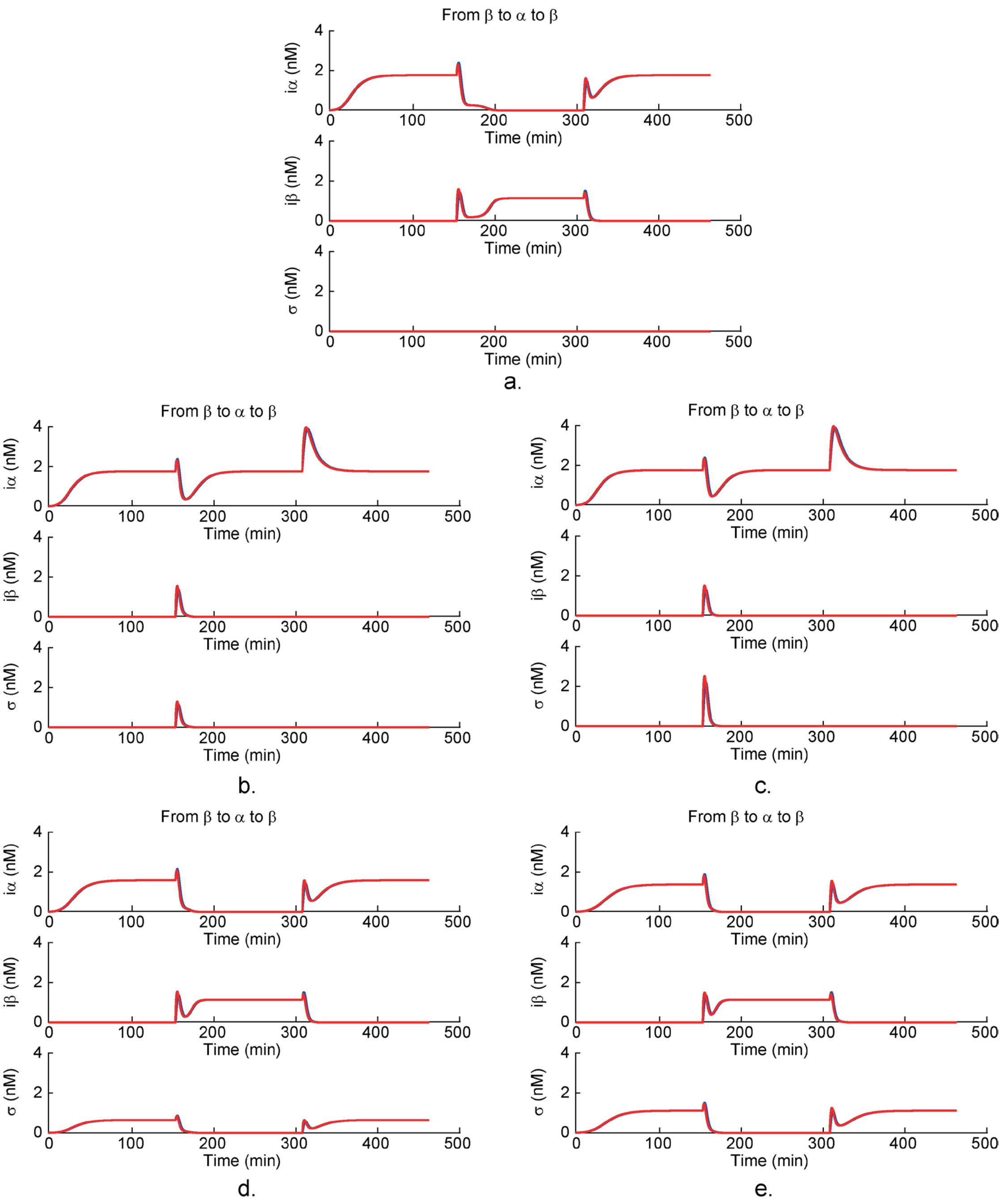

Supplementary Figure 23. Comparison of dynamics of full heuristic model and the reduced model. ***iα***, ***iβ*** and ***σ*** obtained with the full heuristic model (blue) and its steady-state approximations $\mathbf{f}_{i\alpha}$, $\boldsymbol{f}_{i\beta}$, and $\boldsymbol{f}_{\sigma}$ (red) for switches from ***β*** to ***α*** to ***β***. **b.** 10 nM translator at ***α*** ($V_{L\alpha}$ = 0.65 nM min^{-1}). **c.** 20 nM translator at ***α*** ($V_{L\alpha}$ = 1.3 nM min^{-1}). **d.** 10 nM translator at ***β*** ($V_{L\beta}$ = 0.65 nM min^{-1}). **e.** 20 nM translator at ***β*** ($V_{L\beta}$ = 1.3 nM min^{-1}). Switches are initiated at t = 160 min and t = 310 min. Switching from ***α*** to ***β*** is initiated by a ***δ***-pulse and switching from ***β*** to ***α*** is initiated by a ***γ***-pulse. The dynamical behavior of the heuristic model and its reduction are qualitative similar. Using the reduced model separatrices and switching planes are obtained (Supplementary Fig. 26-29). A detailed description of the heuristic model and its steady-state approximations is provided in Supplementary Notes.

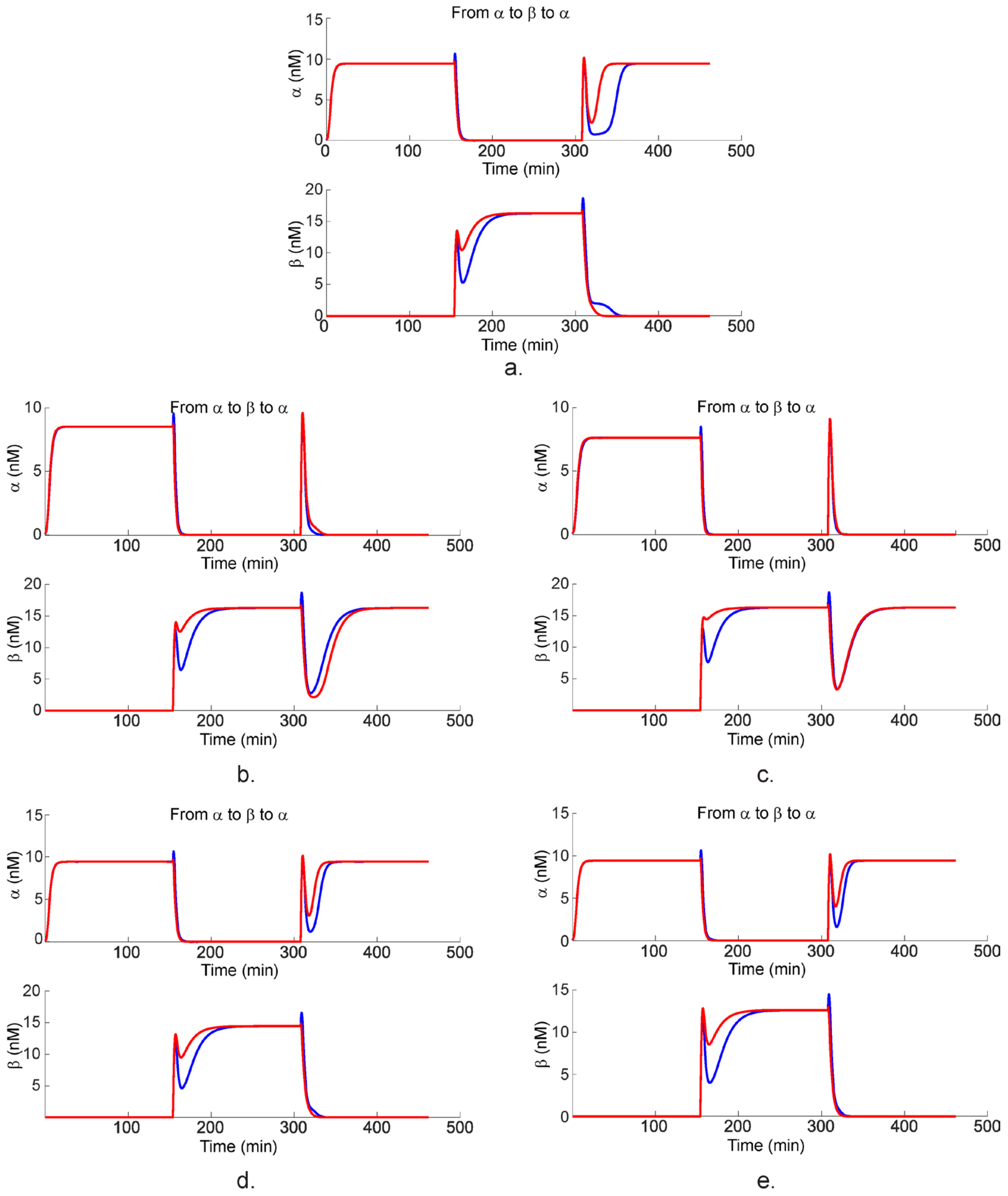

Supplementary Figure 24. Comparison of dynamics of full heuristic model and the reduced model. Comparison of trajectories of the heuristic model (blue) and the reduced model (red) for switches from ***α*** to ***β*** to ***α***. **b.** 10 nM translator at ***α*** ($V_{L\alpha}$ = 0.65 nM min^{-1}). **c.** 20 nM translator at ***α*** ($V_{L\alpha}$ = 1.3 nM min^{-1}). **d.** 10 nM translator at ***β*** ($V_{L\beta}$ = 0.65 nM min^{-1}). **e.** 20 nM translator at ***β*** ($V_{L\beta}$ = 1.3 nM min^{-1}). Switches are initiated at t = 160 min and t = 310 min. Switching from ***α*** to ***β*** is initiated by a ***δ***-pulse and switching from ***β*** to ***α*** is initiated by a ***γ***-pulse. The dynamical behavior of the heuristic model and its reduction are qualitative similar. Using the reduced model separatrices and switching planes are obtained (Supplementary Fig. 26-29). A detailed description of the heuristic model and its steady-state approximations is provided in Supplementary Notes.

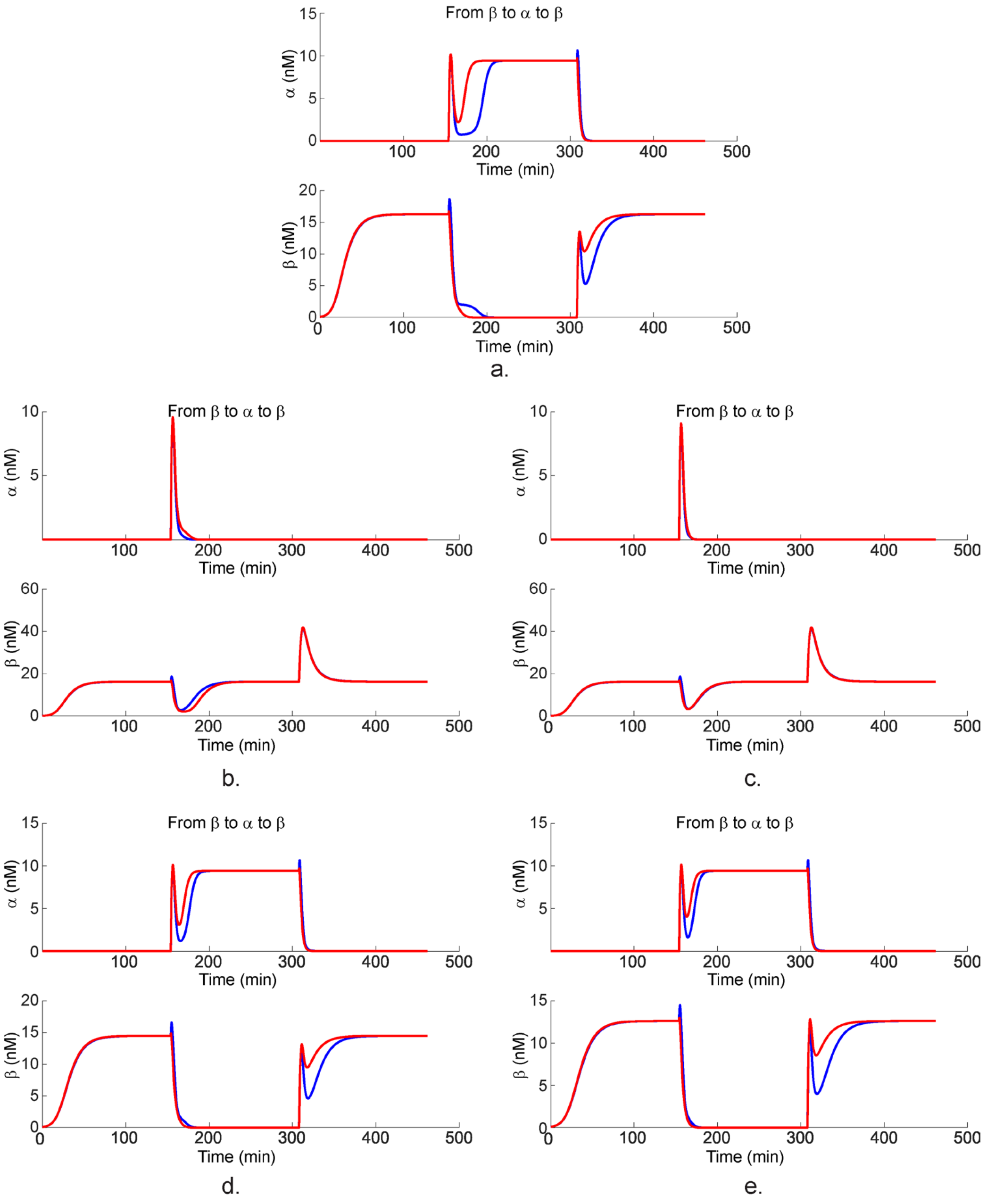

Supplementary Figure 25. Comparison of dynamics of full heuristic model and the reduced model. Comparison of trajectories of the heuristic model (blue) and the reduced model (red) for switches from ***β*** to ***α*** to ***β***. **a.** No translator module. **b.** 10 nM translator at ***α*** ($V_{L\alpha}$ = 0.65 nM min^{-1}). **c.** 20 nM translator at ***α*** ($V_{L\alpha}$ = 1.3 nM min^{-1}). **d.** 10 nM translator at ***β*** ($V_{L\beta}$ = 0.65 nM min^{-1}). **e.** 20 nM translator at ***β*** ($V_{L\beta}$ = 1.3 nM min^{-1}). Switches are initiated at t = 160 min and t = 310 min. Switching from ***α*** to ***β*** is initiated by a ***δ***-pulse and switching from ***β*** to ***α*** is initiated by a γ-pulse. The dynamical behavior of the heuristic model and its reduction are qualitative similar. Using the reduced model separatrices and switching planes are obtained (Supplementary Fig. 26-29). A detailed description of the heuristic model and its steady-state approximations is provided in Supplementary Notes.

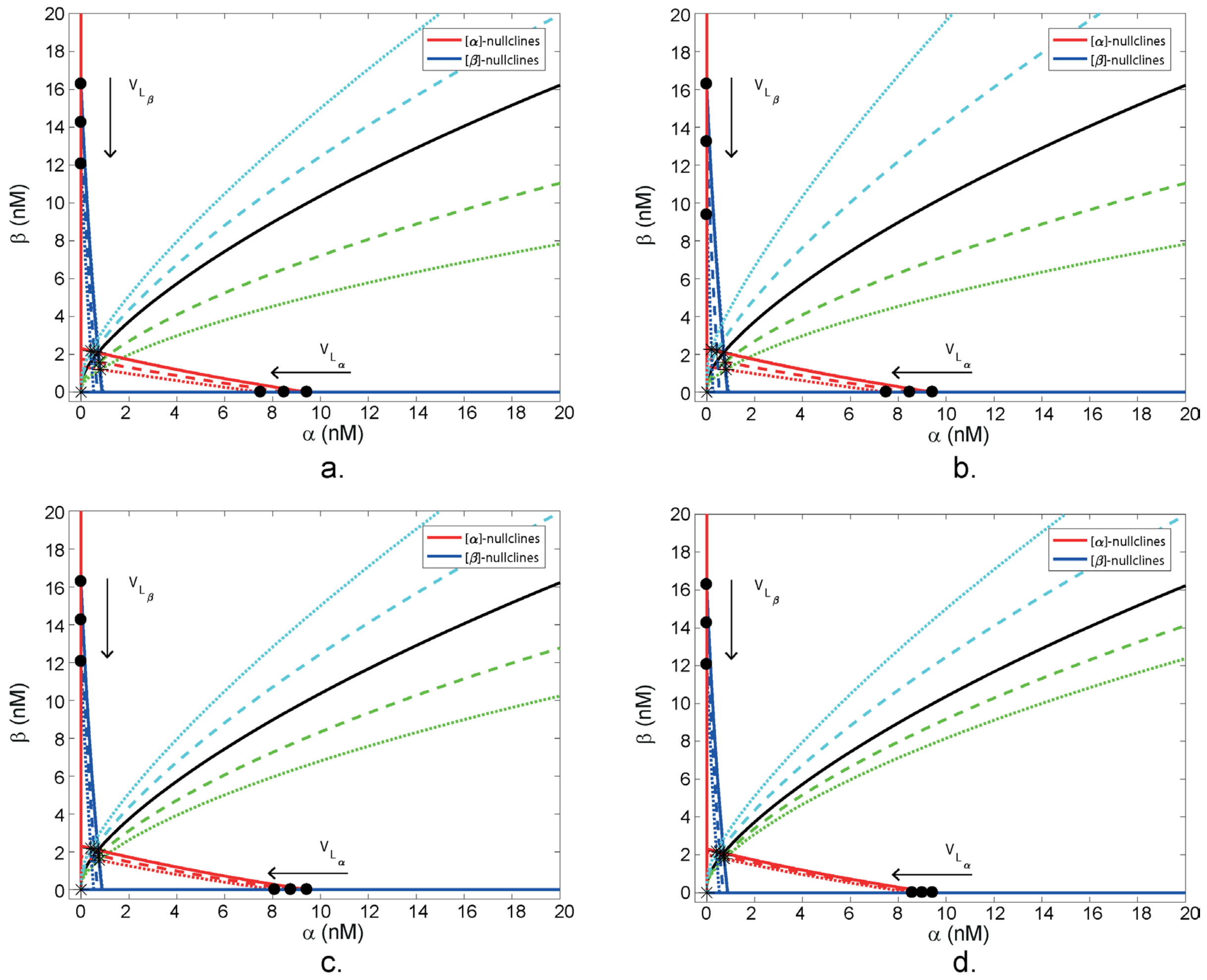

Supplementary Figure 26. Nullclines and seperatrices of the switch without and with translator. Nullclines and separatrices in the (***α***,***β***)-plane for various combinations of $K_{L\alpha}$ and $K_{L\beta}$ were obtained using the reduced model (Supplementary Notes). **a.** $K_{L\alpha}$ = 6 nM, $K_{L\beta}$ = 24 nM **b.** $K_{L\alpha}$ = 6 nM, $K_{L\beta}$ = 12 nM **c.** $K_{L\alpha}$ = 12 nM, $K_{L\beta}$ = 24 nM **d.** $K_{L\alpha}$ = 24 nM, $K_{L\beta}$ = 24 nM. All other parameter are set to their nominal value. Solid lines correspond to absence of translator, dashed line represent presence of translator with load of 10 nM, dotted line represent presence of translator with load of 20 nM. Black line is the seperatrix in absence of translator, green (dashed, dotted) lines are seperatrices for the translator coupled to the ***α***-side of the switch, cyan (dashed, dotted) lines are seperatrices for the translator coupled to the ***β***-side of the switch. Black circles are the (locally) stable steady-states, stars indicate the unstable steady-states. These results show a shift in the nullclines and separatrices when the translator is coupled to the ***α***- or ***β***-side of the switch. Furthermore, the nullclines and separatrices with the translator module coupled show an increased shift with decreasing dissociation constant.

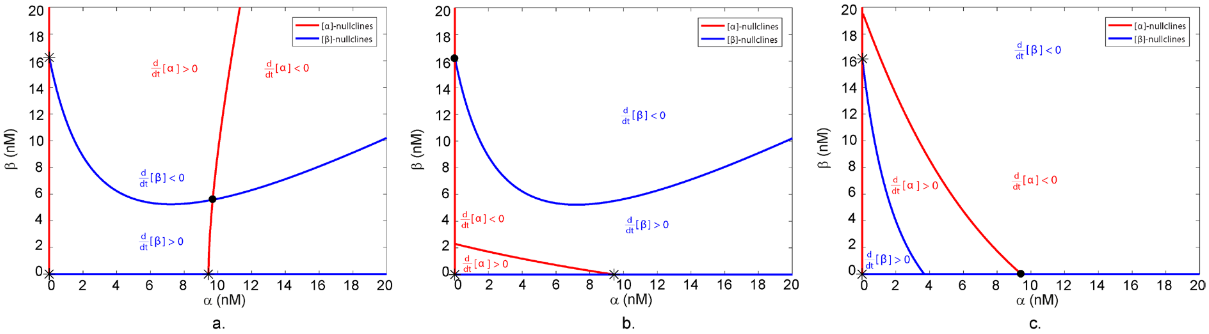

Supplementary Figure 27. Nullcines of the switch without translator. Nullclines of the reduced model (Supplementary Notes) in the (***α***,***β***)-plane for various combinations of λ_α and λ_β. **a.** λ_α = 2, λ_β = 8 **b.** λ_α = 25, λ_β = 8 **c.** λ_α = 6, λ_β = 12. All other parameter are set to their nominal value. Black circles are the (locally) stable steady-states, stars indicate the unstable steady-states. The existence of an unstable positive steady-state, necessary for bistability (Supplementary Notes), is mainly controlled by the inhibition parameters λ_α and λ_β. In particular the unstable positive steady-state can be shown to exist only if both λ_α and λ_β are sufficiently large.

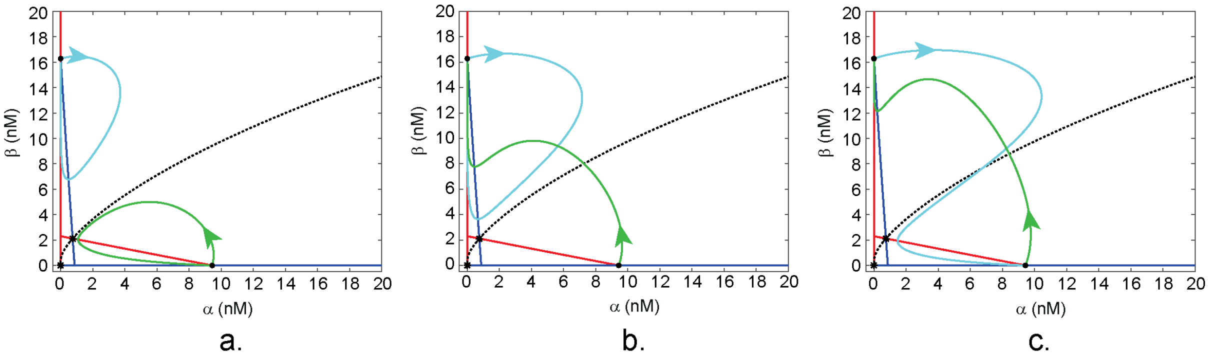

Supplementary Figure 28. **Projection of the trajectories of the switch without translator.** Projection of the trajectories of the reduced model without translator in the (***α***,***β***)-plane with the nullcline of ***α*** in red and the nullcline of ***β*** in blue and the seperatrix indicated by the dashed black line. Green trajectories correspond to case of switching from ***α*** to ***β***, triggered by a ***δ***-pulse with initial amplitude A **a.** A = 10 nM, **b.** A = 20 nM, **c.** A = 30 nM

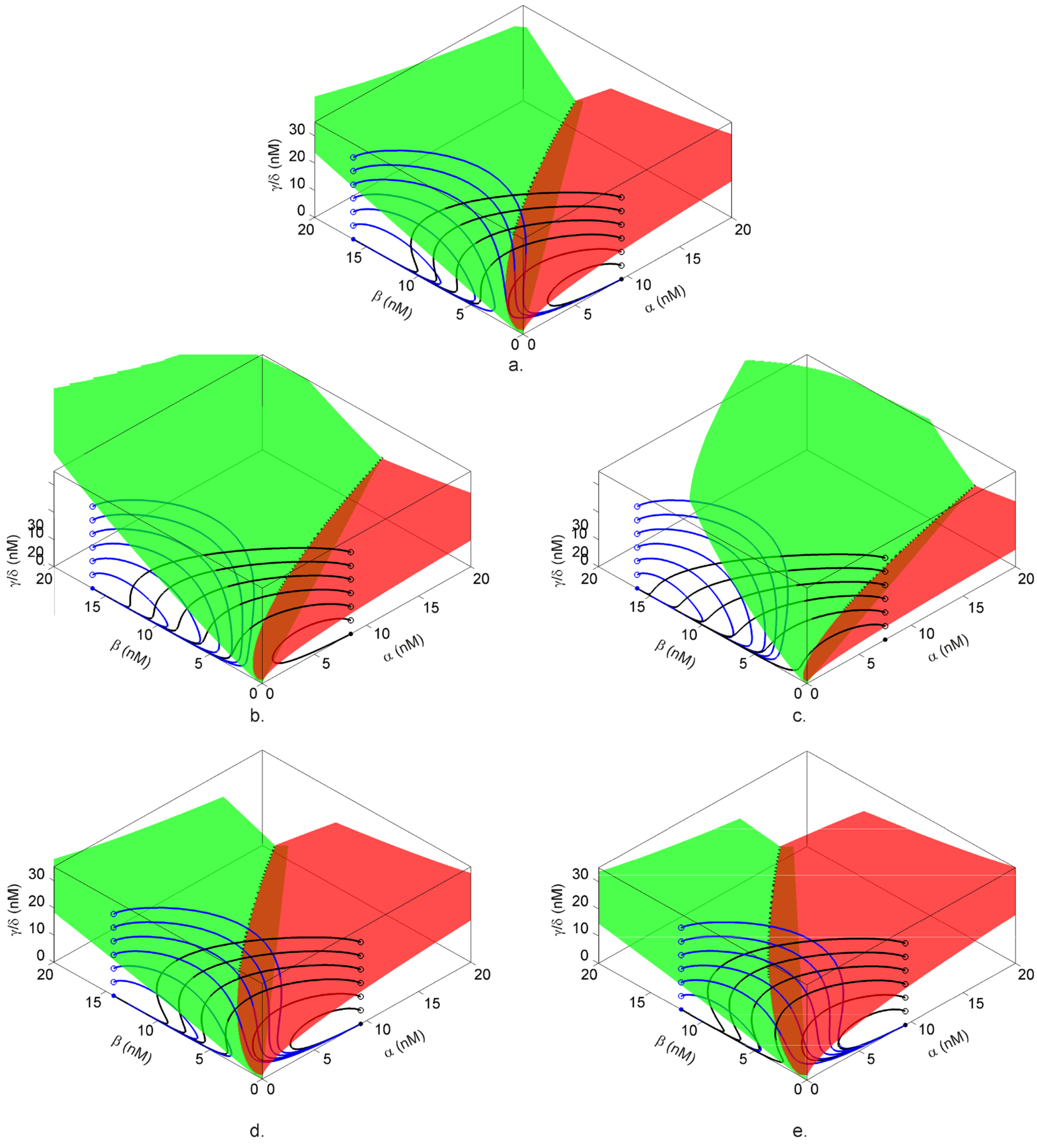

Supplementary Figure 29. **Computed switch-planes without and with translator module.** Computed ***γ***-switch-planes (green) and ***δ***-switch-planes (red) for the switch without translator module and the translator module coupled to ***α*** or ***β***. **a.** Switch planes of switch without translator module. **b.** Switch-planes of switch with 10 nM translator module coupled to ***α*** ($V_{L\alpha}$ = 0.65 nM). **c.** Switch-planes of switch with 20 nM translator module coupled to ***α*** ($V_{L\alpha}$ = 1.3 nM). **d.** Switch-planes of switch with 10 nM translator module coupled to ***β*** ($V_{L\beta}$ = 0.65 nM). **e.** Switch-planes of switch with 20 nM translator module coupled to ***β*** ($V_{L\beta}$ = 1.3 nM). The switch-planes were obtained from the reduced model (Supplementary Notes). Black trajectories corresponding to a (succesful/unsuccesful) switch from ***α*** to ***β***, blue trajectories corresponding to a (succesful/unsuccesful) switch from ***β*** to ***α***.

Black trajectories can not cross the red plane, blue trajectories can not cross the green plane. Open circles indicate the initial conditions and solid circles indicate the final steady-state. The switch-planes were computed with ***γ/δ*** pulses from 5 to 30 nM. Coupling of the translator template to ***β*** results in a shift in the switching plane in favor of the α-state. Contrary, coupling of the translator template to ***α*** results in a shift in the switching plane in favor of the β-state.

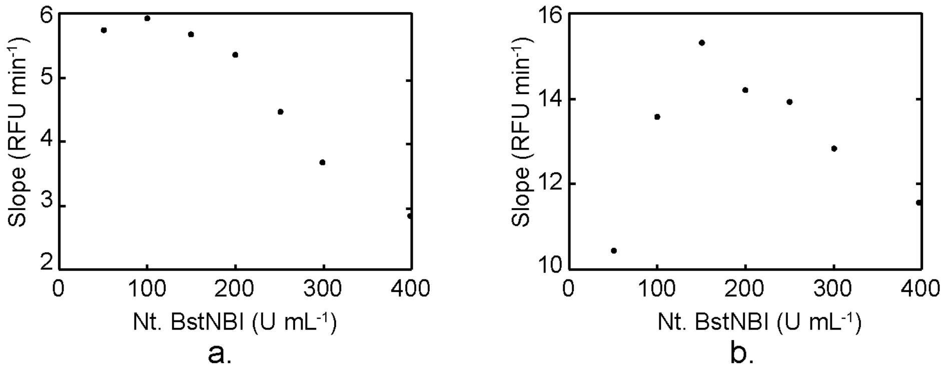

Supplementary Figure 30. **Amplification rate of *α* for a range of nickase.** Experiments were performed for a range of nickase concentration and 20 U/mL Bst. polymerase 2.0 warmstart using 20 nM (a) or 60 nM (b) of autocatalytic template. The amplification rate first increases to an optimum with increasing concentration of nickase after which the amplification rate decreases with increasing concentration of nickase. Furthermore, the optimum ratio of nickase to polymerase is dependent on the concentration of the substrate.

Supplementary Table 1. DNA sequences

		Sequence (5' -> 3')	Length (# bases)	3' modification	5' modification
**INVERTER/Bistable switch**					
Templates	***αtoα***	C*C*A*AGAC***U***CAG-CCAAGACTCAG	22	phosphate	
	βtoβ	A*A*C*AGAC***U***CGA-AACAGACTCGA	22	phosphate	
	αtoiβ	T*T*A*CTCGAAACAGAC-CCAAGACTCAG	26	FAM	
	βtoiα	T*T*A*CTCAGCCAAGAC-AACAGACTCGA	26	DY530	
	γtoα	C*C*A*AGAC***U***CAG-GCATGACTCAT	22	phosphate	
	δtoβ	A*A*C*AGAC***U***CGA-CACTGACTCCT	22	phosphate	
Inputs	***α***	CTGAGTCTTGG	11		
	β	TCGAGTCTGTT	11		
	γ	ATGAGTCATGC	11		
	δ	AGGAGTCAGTG	11		
Inhibitors	***iα***	GTCTTGGCTGAGTAA	15		
	iβ	GTCTGTTTCGAGTAA	15		
**Translator in isolation from upstream and downstream network (Figure 2)**					
Template	***αtoX$_{36}$***	G*T*A*GTAGTTCATTAGTGTCGTTC GTTCACAGTAATA-CCAAGACTCAG	46	phosphate	
Output	***X$_{36}$***	TATTACTGTGAACGAACGACACTAAT GAACTACTAC	36		
Template	***αtoX$_{46}$***	C*G*T*TCGTATGGTAGTAGTTCATT AGTGTCGTTCGTTCACAGTAATA-CCAAGACTCAG	46	phosphate	
Output	***X$_{46}$***	TATTACTGTGAACGAACGACACTAAT GAACTACTACCATACGAACG	36		
Template	***αtoX$_{56}$***	G*C*G*TATTCAGCGTTCGTATGGTA GTAGTTCATTAGTGTCGTTCGTTCAC AGTAATA-CCAAGACTCAG	46	phosphate	
Output	***X$_{56}$***	TATTACTGTGAACGAACGACACTAAT GAACTACTACCATACGAACGCTGAAT ACGC	36		
Reporter	***MB$_X$***	*TATTACTGTGA-*	44	Iowa Black	Cy5

		GTAGTTCATTAGTGTCGTTCGT-*TCACAGTAATA*		RQ-Sp	
**Translator INVERTER (Figure 3)**					
Template	***αtoσ***	T*A*T*TACTGTGAGTAGTTCATTAG TGTC GTTCGTTC-CCAAGACTCAG	46	phosphate	
Output	***σ***	GAACGAACGACACTAATGAACTACT CACAGTAATA	35		
Reporters	***MB$_σ$***	*TATTACTGTGAG*-TAGTTCATTAGTGTCGTTCGT-*CTCACAGTAATA*	45	Iowa Black RQ-Sp	Cy5
**Orthogonal system (Figure 4-6)**					
Templates	***αtoσ***	T*A*T*TACTGTGAGTAGTTCATTAG TGTC GTTCGTTC-CCAAGACTCAG	46	phosphate	
	βtoσ (Fig. 4)	T*A*T*TACTGTGAGTAGTTCATTAG TGTCGTTCGTTC-AACAGACTCGA	46	phosphate	
	βtoε (Fig. 5, 6)	C*A*A*CACAACCCACAACACACCAC CACCGCAACCACCCACCACCAACACC A-AACAGACTCGA	61	phosphate	
Outputs	***σ***	GAACGAACGACACTAATGAACTACT CACAGTAATA	35		
	ξ	TGGTGTTGGTGGTGGGTGGTTGCGG TGGTGGTGTGTTGTGGGTTGTGTTG	50		
Molecular beacons	***MB$_σ$***	*TATTACTGTGAG*-TAGTTCATTAGTGTCGTTCGT-*CTCACAGTAATA*	45	Iowa Black RQ-Sp	Cy5
	MB$_ξ$	*CAACACAACCCA*-CAACACACCACCACCGCAACC-*TGGGTTGTGTTG*	45	Iowa BlackRQ-Sp	ROX
βlac-actuator	***βlac-ODN***	TGTCACCGATGAAACTGTCTA	21	C_6-Amine	
	BLIP-ODN	GTGATGTAGGTGGTAGAGGAA	21		Amine-C_6
	βlacLink- ξ	TTCCTCTACCACCTACATCAC-CAACACAACCCACAACACACCACCAC CGCAACCACCCACCACCAACACCA-TAGACAGTTTCATCGGTGACA	92		
NanoLuc-actuator	***NL-ODN$_{NL}$***	GTGATGTAGGTGGTAGAGGAA	21		Amine

	NLlink- σ	T*T*C*CTCTACCACCTACATCAC-<br>*TATTACTGTGAG*-<br>TAGTTCATTAGTGTCGTTCGT-<br>*CTCACAGTAATA*	66	FAM	

[1] * Indicate phosphorothioate modifications

[2] Complementary sequences are represented by the colors, except for ***iα*** and ***iβ*** with the autocatalytic templates

[3] Underlined sequences represent the nickase recognition site

[4] Replacement of thymine by an uracil is indicated in italic bold

[5] The complementary sequences of the stem of the molecular beacons is denoted in italic

[7] Different domains in the sequences are separated by '-'

Supplementary Table 2. Thermodynamic and kinetic parameters determined in separate experiments

**DNA EQUILIBRIUM DISSOCIATION CONSTANTS**			
	**K_x (nM)**	**T_M (°C)**	**ΔG^o (kcal mol^{-1})**
α/Lα	6	50	-14.3
β/Lβ	24	49	-13.5
iα	0.25	56	-16.3
iβ	0.86	54	-15.6
γ	26	48	-13.4
δ	18	49	-13.7

**DNA ASSOCIATION RATE CONSTANT**
0.13 nM^{-1} min^{-1}

**EXONUCLEASE (10 nM)**		
**V_{max} (nM min^{-1})**	**K_M (nM)**	
27	45	

**REPORTER**	
	**k_{rep} (M^{-1} min^{-1})**
Molecular beacon	5.3 x 10^7

Supplementary Notes

Determination of the thermodynamic dissociation constant of DNA hybridization

The thermodynamic dissociation constants of DNA hybridization were determined from the melting curves (Supplementary Fig. 18 and Supplementary Table 2), obtained using JASCO V-650 spectrophotometer and a 1 cm path length cuvette with a volume of 200 µL. UV absorbance of all possible partial duplexes in the DNA-based network was measured, at a wavelength of 260 nm, as a function of temperature. A temperature gradient of 1°C min^{-1} was used, since melting and cooling profiles were significantly similar at this gradient. The melting curves were converted from absorbance to fraction associated (DNA hybrid) using two baselines (Supplementary Fig. 18a). This curve was used for non-linear least squares analysis using the following equation to obtain the enthalpy and entropy:[4]

$$\theta(T)=1+\frac{1}{2C_0\exp\left(-\frac{\Delta \mathrm{H}^o - T\Delta S^o}{\mathrm{RT}}\right)}\pm\sqrt{\frac{4C_0\exp\left(-\frac{\Delta \mathrm{H}^o - T\Delta S^o}{\mathrm{RT}}\right)+1}{4C_0^2\exp\left(-\frac{2\left(\Delta \mathrm{H}^o - T\Delta S^o\right)}{\mathrm{RT}}\right)}} \quad (1)$$

where θ is the fraction of partially duplex, *T* is the temperature in Kelvin, *R* is the gas constant (kcal mol^{-1} K^{-1}), and C_0 is the initial concentration of the duplex DNA (M) divided by the molarity of water (M). Nonlinear least square optimization was performed using the Matlab routine *lsqnonlin* which uses a subspace trust-region method based on the interior-reflective Newton method. The enthalpy (kcal mol^{-1} K^{-1}) and entropy (kcal mol^{-1}) obtained from this fitting were used to determine the standard Gibb's free energy (kcal mol^{-1}) of DNA hybridization (from which the thermodynamic dissociation constant can be calculated) at a temperature of 42 °C:

$$\Delta G^o = \Delta H^o - T\Delta S^0 \quad (2)$$

The thermodynamic binding constants of the measured hybrids are shown in Supplementary Table 2.

Determination of the forward rate constant of DNA hybridization

To characterize the forward rate constant of DNA hybridization (k_a) experiments were performed using the stopped-flow device (BioLogic, MOS-500 spectrophotometer equipped with a SFM-2000 mixing system) with a dead time of 0.25 ms in absorbance mode (Supplementary Fig. 19 and Supplementary Table 2). Multiple experiments (>6) were performed in which absorbance was recorded over time at 42 °C after mixing 1 µM of primer *β* to 1 µM of template *δ*to*β* both in 1 x TE

buffer complemented with 0.06 M Na^+, 0.008 M Mg^{2+} and preheated at 42 °C. This yielded final concentrations of 500 nM primer *β* and 500 nM of template *δ*to*β*. The raw data was subtracted by the absorbance at time = 0. Thereafter, the absorbance was converted to concentration of duplex (dsDNA) by assuming the reaction was equilibrated in 5 seconds and the experimentally determined thermodynamic dissociation constant (Supplementary Table 2 and *vide supra*). Finally, the mean of the multiple experiments was determined for further analysis. To obtain the second-order rate constant (k_a) non-linear least squares multiple-curve fitting was performed (black) using the Matlab routine lsqnonlin with a subspace trust-region method based on the interior-reflective Newton method. The hybridization association equilibrium constant (K_β) was determined experimentally (*vide supra*) and was fixed during the non-linear least-square analysis (Supplementary Fig. 19).

Determination of the kinetics of exonuclease

To characterize the kinetics of the exonuclease experiments were performed in which 1.5, 2 and 2.5 µM of a ssDNA strand were added to eppendorfs (white real-time PCR tube strips, Eppendorf) in minimal volumes (Supplementary Fig. 20 and Supplementary Table 2). Then, master mix with the DNA intercalating dye EvaGreen (2x) and 5 nM exonuclease ttRecJ was added leading to a final volume of 20 µL. After all components were assembled the mixtures are vortexed and spinned down and subsequently the eppendorfs were placed in the Polymerase Chain Reaction (PCR) system (CFX96 from Bio-Rad) which was prewarmed at 42°C with a lid temperature of 70°C to prevent condensation. The relative fluorescence units (RFU) were measured over cycles of 12 seconds. For every datacurve the same analysis procedure was followed. The very first part (5 min) of the RFU versus time curve often contains an under or overshoot (*vide infra*), caused by equilibration effects. When this was observed the initial data was removed, and an estimate of these datapoints was made by extrapolation, i.e. by performing linear fitting to the first part of the raw data curve an estimate could be made of the RFU of the first datapoints. The RFU at the timepoint at which its values became constant, corresponding to the timepoint at which all non-protected primer was degraded by exonuclease, was subtracted from the datacurve, so that at $t = t_{end}$ an RFU of zero was obtained. Then, the RFU was converted to the concentration of ssDNA by dividing the RFU with a factor obtained by dividing the initial RFU by the initial concentration of degradable ssDNA. Using the datacurve obtained from the experiment without competitor (Supplementary Fig. 20a), the Michaelis-Menten parameters, V_{max} and K_m, were determined by non-least squares fitting of the integrated Michaelis-Menten equation:

$$V_{\max} t = [S_0] - [S(t)] + K_M \ln\left(\frac{[S_0]}{S(t)}\right) \tag{3}$$

With $[S_0]$ the initial concentration of oligonucleotide, [S(t)] the concentration of oligonucleotide at a certain time t, V_{max} the maximal rate of enzyme and K_m the Michaelis-Menten constant. To determine the parameters, V_{max} and K_m non-linear least squares curve fitting was performed using the Levenberg-Marquardt algorithm. To prevent entrapment in local minima of the cost function, Latin Hypercube sampling was used to create twenty initial parameter vectors, with values in between an interval of 0.01 and 100 times of the expected parameter value. From these twenty initial parameter values the parameter values corressponding to the lowest residual sum of squares was selected.

Determination of the kinetics of the molecular beacon

To characterize the kinetics of the molecular beacon, experiments in triplo were performed (Supplementary Fig. 21 and Supplementary Table 2) in which the molecular beacon in master mix without enzymes was added to a cuvette which was preheated at 42 °C in a fluorescence spectrophotometer (Carry Eclipse equipped with a Varian Peltier Multicell Holder and a Cary Temperature Controller). Subsequently, the fluorescence of the molecular beacon was measured to obtain the baseline fluorescence. Thereafter, DNA strand ***σ*** was added giving a volume of 120 µL after which the mixture was suspended and measurement started at 42 °C. Data handling was done for each single curve in the same way. First, the baseline fluorescence was determined by taking the mean of a one minute measurement of the fluorescence from the molecular beacon. After this, the fluorescence from the measurement was subtracted by this baseline value. Subsequently, the missing points due to suspending (10-15 seconds) were estimated by extrapolation. Thereafter, the fluorescence was converted to concentration of opened beacon by assuming the reaction was equilibrated in 18 minutes. An ODE model based on the bimolecular reaction model of DNA strand displacement was developed to describe the kinetics of this step.[5] To obtain the second-order rate constant (k_{rep}) of the toehold mediated strand displacement reaction non-linear least-square optimization of the experimental kinetic traces (depicted in Supplementary Fig. 21b) to the mathematical model was performed. The Matlab routine lsqnonlin with a subspace trust-region method based on the interior-reflective Newton method was applied, yielding a k_{rep} of 5.3 x 10^7 M^{-1} min^{-1}.

Mathematical model of Bistable Switch: A heuristic model

Following [1], we assume that the activation of a primer y on a template T and an activator x

$$x + T \underset{k_{-1}}{\overset{k_1}{\rightleftharpoons}} xT \xrightarrow{k_2} x + y + T$$

can be described by the Michaelis-Menten derivation:

$$\frac{d}{dt}[y] = \frac{V[x]}{K + [x]}; K = \frac{k_{-1} + k_2}{k_1} \tag{4}$$

where V is proportional to the total concentration of template. Furthermore, the second step with reaction rate k_2 comprises two enzymatic reactions including polymerization and nicking. Therefore, we assume $k_2 << k_{-1}$ and K becomes the dissociation constant of x on T which can be experimentally determined (*vide infra*). Here the square bracket notation [ ] is used to denote the concentration of the corresponding species. (We remark that t is an independent variable that only resembles time.) If iy is a competitive inhibitor acting on y, then

$$\frac{d}{dt}[y] = \frac{V[x]}{K + [x] + \lambda_y [iy]} \tag{5}$$

where constant λ_y roughly depends on the dissociation constant of iy and the dissociation constant of x.

The degradation of a primer x,

$$x + E \rightleftharpoons xE \longrightarrow E$$

will be described by a competitive Michaelis-Menten mechanism:

$$\frac{d}{dt}[x] = -\frac{V_{exo}[x]}{K_{M,exo}\left(1 + [x]/K_{M,exo} + [y]/K_{M,exo} + \ldots\right)} \tag{6}$$

with the Michaelis-Menten constant $K_{M,exo}$ as experimentally determined ($videinfra$). The parameter V_{exo} was estimated to be lower as experimentally determined in isolation, since sequestration of exonuclease by the templates is present. We manually adapted this value.

Bistable switch

The bistable switch consists of six primers:

- γ, an activator of α;
- δ, an activator of β;
- α, an activator of both α (autocatalysis) and $i\beta$;
- β, an activator of both β (autocatalysis) and $i\alpha$;
- $i\alpha$, an competitive inhibitor of α (autocatalysis);
- $i\beta$, an competitive inhibitor of β (autocatalysis).

Furthermore, each primer degrades via exonuclease.

Using heuristics ($videsupra$) we propose the following dynamical model of the bistable switch:

$$\begin{aligned}
\frac{d}{dt}[\gamma] &= -\frac{V_{exo}[\gamma]}{K_{exo} + [\gamma] + [\delta] + [\alpha] + [\beta] + [i\alpha] + [i\beta]}, \\
\frac{d}{dt}[\delta] &= -\frac{V_{exo}[\delta]}{K_{exo} + [\gamma] + [\delta] + [\alpha] + [\beta] + [i\alpha] + [i\beta]}, \\
\frac{d}{dt}[\alpha] &= \frac{V_\gamma[\gamma]}{K_\gamma + [\gamma]} + \frac{V_\alpha[\alpha]}{K_\alpha + [\alpha] + \lambda_\alpha[i\alpha]} - \frac{V_{exo}[\alpha]}{K_{exo} + [\gamma] + [\delta] + [\alpha] + [\beta] + [i\alpha] + [i\beta]}, \\
\frac{d}{dt}[\beta] &= \frac{V_\delta[\delta]}{K_\delta + [\delta]} + \frac{V_\beta[\beta]}{K_\beta + [\beta] + \lambda_\beta[i\beta]} - \frac{V_{exo}[\beta]}{K_{exo} + [\gamma] + [\delta] + [\alpha] + [\beta] + [i\alpha] + [i\beta]}, \\
\frac{d}{dt}[i\alpha] &= \frac{V_{i\alpha}[\beta]}{K_{i\alpha} + [\beta]} - \frac{V_{exo}[i\alpha]}{K_{exo} + [\gamma] + [\delta] + [\alpha] + [\beta] + [i\alpha] + [i\beta]}, \\
\frac{d}{dt}[i\beta] &= \frac{V_{i\beta}[\alpha]}{K_{i\beta} + [\alpha]} - \frac{V_{exo}[i\beta]}{K_{exo} + [\gamma] + [\delta] + [\alpha] + [\beta] + [i\alpha] + [i\beta]}.
\end{aligned} \tag{7}$$

Switch with translator

We describe the model of the translator for the case that it is coupled to the α-side of the switch. Coupling to the β-side of the switch is done analogously.

The chemical scheme of the translator is as follows:

$$\alpha + T_L\sigma \rightleftharpoons \alpha T_L\sigma \xrightarrow{\text{pol. SD}} \alpha\sigma T_L + \sigma$$

$$\alpha\sigma T_L \xrightarrow{\text{nic.}} \alpha T_L\sigma \ .$$

Furthermore, σ degrades via exonuclease

$$\sigma \xrightarrow{\text{exo.}} \emptyset$$

and binds to a reporter

$$\sigma + MB \longrightarrow \sigma MB.$$

We model the activation of σ via α again via a Michaelis-Menten mechanism:

$$\tfrac{d}{dt}[\sigma] = \frac{V_{L_\alpha}[\alpha]}{K_{L_\alpha} + [\alpha]} \tag{8}$$

The activation of σ changes the concentration of α by:

$$\tfrac{d}{dt}[\alpha] = -\frac{V_{L_\alpha}[\alpha]}{K_{L_\alpha} + [\alpha]} + k_\alpha[\theta] \tag{9}$$

where the first term at the right-hand-side represents the amount of α that is used by the translator for the production of σ, and the second term accounts for the reproduction of α due to the dissociation of α from the nicked translator module. The rate of reproduction is given by θ representing the concentration of nicked translator module, linearly scaled with the dissociation rate constant of α. Furthermore, the ordinary differential equation of θ is given by:

$$\tfrac{d}{dt}[\theta] = \rho\frac{V_{L_\alpha}[\alpha]}{K_{L_\alpha} + [\alpha]} - k_\alpha[\theta] \tag{10}$$

where k_α models the dissociaton rate constant of α and ρ models the fraction of translator module being in the nicked state. The dissociation rate constant of α is determined by the equilibrium dissociation constant $K_{L\alpha}$ as the association rate constant is invariable for primers with lengths exceeding five bases.[4] The constant depends on the timescale of nicking the duplex relative to the timescale of the polymerase strand displacement reaction. In the extreme case of $\rho = 1$ the equilibrium of the two states of the translator module is shifted to the nicked state (minimal inherent retroactivity) and, hence, the amount of α reproduced depends on $K_{L\alpha}$. In the other extreme case $\rho = 0$ no α is reproduced (maximal inherent retroactivity) independent on $K_{L\alpha}$. Note that for being at steady-state it is required that the rate of polymerase is identical to the rate of nickase, which implies that ρ depends, to a large extend, on the reaction rates of polymerase and nickase.

The binding of σ to the reporter MB is assumed to be governed by mass-action-kinetics and can be described by the system of ODEs

$$\begin{aligned} \tfrac{d}{dt}[\sigma] &= -k_{rep}[\sigma][MB] \\ \tfrac{d}{dt}[MB] &= -k_{rep}[\sigma][MB] \\ \tfrac{d}{dt}[\sigma MB] &= k_{rep}[\sigma][MB] \end{aligned} \tag{11}$$

with rate constant k_{rep} which can be experimentally determined (*vide infra*).

We then model the bistable switch with translator at the α-side and reporter by the systems of ODEs:

$$\begin{aligned}
\tfrac{d}{dt}[\gamma] &= -\frac{V_{exo}[\gamma]}{K_{exo}+[\gamma]+[\delta]+[\alpha]+[\beta]+[i\alpha]+[i\beta]+[\sigma]},\\
\tfrac{d}{dt}[\delta] &= -\frac{V_{exo}[\delta]}{K_{exo}+[\gamma]+[\delta]+[\alpha]+[\beta]+[i\alpha]+[i\beta]+[\sigma]},\\
\tfrac{d}{dt}[\alpha] &= \frac{V_\gamma[\gamma]}{K_\gamma+[\gamma]}+\frac{V_\alpha[\alpha]}{K_\alpha+[\alpha]+\lambda_\alpha[i\alpha]}-\frac{V_{exo}[\alpha]}{K_{exo}+[\gamma]+[\delta]+[\alpha]+[\beta]+[i\alpha]+[i\beta]+[\sigma]}\\
&\quad -\frac{V_{L_\alpha}[\alpha]}{K_{L_\alpha}+[\alpha]}+k_\alpha[\theta],\\
\tfrac{d}{dt}[\beta] &= \frac{V_\delta[\delta]}{K_\delta+[\delta]}+\frac{V_\beta[\beta]}{K_\beta+[\beta]+\lambda_\beta[i\beta]}-\frac{V_{exo}[\beta]}{K_{exo}+[\gamma]+[\delta]+[\alpha]+[\beta]+[i\alpha]+[i\beta]+[\sigma]},\\
\tfrac{d}{dt}[i\alpha] &= \frac{V_{i\alpha}[\beta]}{K_{i\alpha}+[\beta]}-\frac{V_{exo}[i\alpha]}{K_{exo}+[\gamma]+[\delta]+[\alpha]+[\beta]+[i\alpha]+[i\beta]+[\sigma]},\\
\tfrac{d}{dt}[i\beta] &= \frac{V_{i\beta}[\alpha]}{K_{i\beta}+[\alpha]}-\frac{V_{exo}[i\beta]}{K_{exo}+[\gamma]+[\delta]+[\alpha]+[\beta]+[i\alpha]+[i\beta]+[\sigma]},\\
\tfrac{d}{dt}[\sigma] &= \frac{V_{L_\alpha}[\alpha]}{K_{L_\alpha}+[\alpha]}-\frac{V_{exo}[\sigma]}{K_{exo}+[\gamma]+[\delta]+[\alpha]+[\beta]+[i\alpha]+[i\beta]+[\sigma]}-k_{rep}[\sigma][MB],\\
\tfrac{d}{dt}[\theta] &= \rho\frac{V_{L_\alpha}[\alpha]}{K_{L_\alpha}+[\alpha]}-k_\alpha[\theta],\\
\tfrac{d}{dt}[MB] &= -k_{rep}[\sigma][MB],\\
\tfrac{d}{dt}[\sigma MB] &= k_{rep}[\sigma][MB].
\end{aligned} \tag{12}$$

Likewise, the model of the bistable switch with translator at the β-side and reporter is given by the systems of ODEs:

$$\begin{aligned}
\tfrac{d}{dt}[\gamma] &= -\frac{V_{exo}[\gamma]}{K_{exo}+[\gamma]+[\delta]+[\alpha]+[\beta]+[i\alpha]+[i\beta]+[\sigma]},\\
\tfrac{d}{dt}[\delta] &= -\frac{V_{exo}[\delta]}{K_{exo}+[\gamma]+[\delta]+[\alpha]+[\beta]+[i\alpha]+[i\beta]+[\sigma]},\\
\tfrac{d}{dt}[\alpha] &= \frac{V_\gamma[\gamma]}{K_\gamma+[\gamma]}+\frac{V_\alpha[\alpha]}{K_\alpha+[\alpha]+\lambda_\alpha[i\alpha]}-\frac{V_{exo}[\alpha]}{K_{exo}+[\gamma]+[\delta]+[\alpha]+[\beta]+[i\alpha]+[i\beta]+[\sigma]}\\
\tfrac{d}{dt}[\beta] &= \frac{V_\delta[\delta]}{K_\delta+[\delta]}+\frac{V_\beta[\beta]}{K_\beta+[\beta]+\lambda_\beta[i\beta]}-\frac{V_{exo}[\beta]}{K_{exo}+[\gamma]+[\delta]+[\alpha]+[\beta]+[i\alpha]+[i\beta]+[\sigma]}\\
&\quad -\frac{V_{L_\beta}[\beta]}{K_{L_\beta}+[\beta]}+k_\beta[\theta],\\
\tfrac{d}{dt}[i\alpha] &= \frac{V_{i\alpha}[\beta]}{K_{i\alpha}+[\beta]}-\frac{V_{exo}[i\alpha]}{K_{exo}+[\gamma]+[\delta]+[\alpha]+[\beta]+[i\alpha]+[i\beta]+[\sigma]},\\
\tfrac{d}{dt}[i\beta] &= \frac{V_{i\beta}[\alpha]}{K_{i\beta}+[\alpha]}-\frac{V_{exo}[i\beta]}{K_{exo}+[\gamma]+[\delta]+[\alpha]+[\beta]+[i\alpha]+[i\beta]+[\sigma]},\\
\tfrac{d}{dt}[\sigma] &= \frac{V_{L_\beta}[\beta]}{K_{L_\beta}+[\beta]}-\frac{V_{exo}[\sigma]}{K_{exo}+[\gamma]+[\delta]+[\alpha]+[\beta]+[i\alpha]+[i\beta]+[\sigma]}-k_{rep}[\sigma][MB],\\
\tfrac{d}{dt}[\theta] &= \rho\frac{V_{L_\beta}[\beta]}{K_{L_\beta}+[\beta]}-k_\beta[\theta],\\
\tfrac{d}{dt}[MB] &= -k_{rep}[\sigma][MB],\\
\tfrac{d}{dt}[\sigma MB] &= k_{rep}[\sigma][MB].
\end{aligned} \tag{13}$$

Steady-states

Let us consider the bistable switch without translator. We perform a steady-state analysis, enabling the selection of parameters for the bistable switch which could not be measured in separate experiments. Clearly, at steady-state $[\gamma] = 0$ nM and $[\delta] = 0$ nM (as $[\gamma]$ and $[\delta]$ only degrade). Furthermore, we are only interested in steady-state solutions of the form:

- SS1: $[\alpha] > 0$ and $[\beta] = 0$;
- SS2: $[\alpha] = 0$ and $[\beta] > 0$.

Note that $[i\alpha] > 0$ if and only if $[\beta] > 0$, and $[i\beta] > 0$ if and only if $[\alpha] > 0$. Then we obtain for SS1:

$$[i\beta] = \frac{V_{i\beta}[\alpha]^2 + K_{exo}V_{i\beta}[\alpha]}{K_{i\beta}V_{exo} + (V_{exo} - V_{i\beta})[\alpha]} = \frac{V_{exo}K_\alpha - V_\alpha K_{exo} + (V_{exo} - V_\alpha)[\alpha]}{V_\alpha} \tag{14}$$

Letting

$$V_{exo} > V_{i\beta}, \quad V_{exo} > V_\alpha, \quad V_{exo}K_\alpha > V_\alpha K_{exo},$$

we ensure that $[i\beta] > 0$ for $[\alpha] > 0$. Likewise, for SS2 we find

$$[i\alpha] = \frac{V_{i\alpha}[\beta]^2 + K_{exo}V_{i\alpha}[\beta]}{K_{i\alpha}V_{exo} + (V_{exo} - V_{i\alpha})[\beta]} = \frac{V_{exo}K_\beta - V_\beta K_{exo} + (V_{exo} - V_\beta)[\beta]}{V_\beta} \tag{15}$$

such that for

$$V_{exo} > V_{i\alpha}, \quad V_{exo} > V_\beta, \quad V_{exo}K_\beta > V_\beta K_{exo},$$

we have $[i\alpha] > 0$ if $[\beta] > 0$. Inequalities:

$$V_{exo} > V_j, \quad j = \alpha, \beta, i\alpha, i\beta$$

ensure that solutions are bounded in forward time. Furthermore, it is interesting to note that

$$V_{exo}K_\alpha > V_\alpha K_{exo},$$
$$V_{exo}K_\beta > V_\beta K_{exo},$$

imply that the trivial steady-state solution

$$([\alpha], [\beta], [i\alpha], [i\beta]) = (0, 0, 0, 0) \tag{16}$$

is unstable. (This can easily be verified by determining the Jacobian matrix around this steady-state). We remark that there also exists an other steady-state solution with $[\alpha] > 0$ and $[\beta] > 0$. This steady-sate can be shown to be unstable when λ_α and λ_β are sufficiently large (Supplementary Fig. 27).

Numerical simulations

Given the constraints on the parameters that are obtained with the steady-state analysis we choose the remaining parameters such that there is a good qualitative correspondence between numerical simulations and experiments. Initial conditions are chosen as

$$\begin{array}{llll} [\alpha](t_0) = 0, & [\beta](t_0) = 0, & [i\alpha](t_0) = 0, & [i\beta](t_0) = 0, \\ [\sigma](t_0) = 0, & [\theta](t_0) = 0, & [\sigma MB](t_0) = 0, & [MB](t_0) = MB_0, \end{array}$$

and

$$[\gamma](t_0) \in \{0,\ A\}, \quad [\delta](t_0) \in \{0,\ A\},$$

with A being the amplitude of the stimulus (concentrations of $[\gamma]$ or $[\delta]$ at initiation of a switch). For the bistable switch we set $MB_0 = 100$ nM and $A = 30$ nM, which correspond to the values used in the experiments. Furthermore we let

$$V_{L_\alpha}, V_{L_\beta} \in \{0, 10, 20\},$$

again in correspondence with the values used in the experiments. We found that parameters

$$\begin{array}{lll} V_\gamma = 8.5 \text{ nM min}^{-1}, & V_{i\alpha} = 1.6 \text{ nM min}^{-1}, & V_{exo} = 23.4 \text{ nM min}^{-1}, \\ V_\delta = 7.2 \text{ nM min}^{-1}, & V_\beta = 15.0 \text{ nM min}^{-1}, & \lambda_\beta = 28, \\ V_\alpha = 6.5 \text{ nM min}^{-1}, & V_{i\beta} = 0.8 \text{ nM min}^{-1}, & \lambda_\alpha = 25, \end{array}$$

and $\rho \in [0,\ 0.4]$ yield the desired qualitative match with experiments (Figure 4 and Supplementary Fig. 5).

A reduced model

In this section we present a reduced model based on our heuristic model. To reduce the model we

- ignore the dynamics of the reporter (by setting either $k_{rep} = 0$ nM min^{-1} or $MB_0 = 0$ nM);
- assume that $[i\alpha]$, $[i\beta]$, $[\sigma]$ and $[\theta]$ are instantly at steady-state.

The first condition is reasonable as the reporter has minor influence on the occurrence of a switch. (In particular, note that $[MB]$ is only "consumed" such that after some switches $[MB] = 0$.) The second assumptions are made without chemical nor mathematical justification. One of the main reasons for reduction is visualization of switch planes in $\mathbb{R}^3_+$. However, from numerical simulations we observe that the steady-state assumption produces reasonably accurate results (Supplementary Fig. 22-23). Furthermore, as shown in Supplementary Fig. 24-25, the dynamical behavior of the heuristic model and its reduction are qualitative similar.

Denoting by:

$$\begin{aligned}
[i\alpha] &= f_{i\alpha}([\alpha],[\beta]) \\
[i\beta] &= f_{i\beta}([\alpha],[\beta]) \\
[\sigma] &= f_{\sigma}([\alpha],[\beta]) \\
[\theta] &= f_{\theta}([\alpha],[\beta])
\end{aligned}$$

the steady-states solutions of $[i\alpha]$, $[i\beta]$, $[\sigma]$ and $[\theta]$ as function of $[\alpha]$ and $[\beta]$, we obtain the reduced model:

$$\begin{aligned}
\tfrac{d}{dt}[\gamma] &= -\frac{V_{exo}[\gamma]}{K_{exo} + [\gamma] + [\delta] + [\alpha] + f_{i\alpha}([\alpha],[\beta]) + f_{i\beta}([\alpha],[\beta]) + f_{\sigma}([\alpha],[\beta])}, \\
\tfrac{d}{dt}[\delta] &= -\frac{V_{exo}[\delta]}{K_{exo} + [\gamma] + [\delta] + [\alpha] + f_{i\alpha}([\alpha],[\beta]) + f_{i\beta}([\alpha],[\beta]) + f_{\sigma}([\alpha],[\beta])}, \\
\tfrac{d}{dt}[\alpha] &= \frac{V_{\gamma}[\gamma]}{K_{\gamma} + [\gamma]} + \frac{V_{\alpha}[\alpha]}{K_{\alpha} + [\alpha] + \lambda_{\alpha} f_{i\alpha}([\alpha],[\beta])} - (1-\rho)\frac{V_{L_\alpha}[\alpha]}{K_{L_\alpha} + [\alpha]} \\
&\quad - \frac{V_{exo}[\alpha]}{K_{exo} + [\gamma] + [\delta] + [\alpha] + [\beta] + f_{i\alpha}([\alpha],[\beta]) + f_{i\beta}([\alpha],[\beta]) + f_{\sigma}([\alpha],[\beta])}, \\
\tfrac{d}{dt}[\beta] &= \frac{V_{\delta}[\delta]}{K_{\delta} + [\delta]} + \frac{V_{\beta}[\beta]}{K_{\beta} + [\beta] + \lambda_{\beta} f_{i\beta}([\alpha],[\beta])} - (1-\rho)\frac{V_{L_\beta}[\beta]}{K_{L_\beta} + [\beta]} \\
&\quad - \frac{V_{exo}[\beta]}{K_{exo} + [\gamma] + [\delta] + [\alpha] + [\beta] + f_{i\alpha}([\alpha],[\beta]) + f_{i\beta}([\alpha],[\beta]) + f_{\sigma}([\alpha],[\beta])},
\end{aligned} \tag{17}$$

where $V_{L_\alpha} V_{L_\beta} = 0$ (which implies that the translator is coupled to at most one side of the switch).

The dynamics of the reduced model will be studied in detail, which provides valuable insights in the role of specific parameters in both the reduced model and the full model.

Nullclines

Let us consider the dynamics of the reduced model with $[\gamma] = 0$ nM and $[\delta] = 0$ nM. We first determine the $[\alpha]$-nullclines and $[\beta]$-nullclines, i.e. the set set of points at which $\frac{d}{dt}[\alpha] = 0$ and $\frac{d}{dt}[\beta] = 0$, respectively. Obviously, the points of intersection of the $[\alpha]$-nullclines and $[\beta]$-nullclines define the steady-state solutions of the reduced model. Note that

$$[\alpha] = 0 \ \Rightarrow \tfrac{d}{dt}[\alpha] = 0 \quad \text{and} \quad [\beta] = 0 \ \Rightarrow \tfrac{d}{dt}[\beta] = 0,$$

such that $([\alpha],[\beta]) = (0,0)$ is a steady-state solution. Furthermore, note that steady-state solutions of the reduced model coincide with (the $[\alpha]$ and $[\beta]$ part of) the steady-state solutions of the full model. Supplementary Fig. 26 shows the nullclines depending on coupling of the translator. In all these plots

- $\frac{d}{dt}[\alpha] < 0$ for point above the red nullclines;
- $\frac{d}{dt}[\alpha] > 0$ for point below the red nullclines;

and

- $\frac{d}{dt}[\beta] < 0$ for point right of the blue nullclines;
- $\frac{d}{dt}[\beta] > 0$ for point left the blue nullclines.

As such it follows that an intersection of the red and blue nullclines is a point with $[\alpha] > 0$ and $[\beta] > 0$ (i.e. the existence of a positive steady-state of the saddle type is necessary for bistability). The existence of this unstable positive steady-state is mainly controlled by the inhibition parameters λ_α and λ_β. In particular the unstable positive steady-state can be shown to exist only if both λ_α and λ_β are sufficiently large. See Supplementary Fig. 27 for some examples for which the bistable switch does not work because of improper values of λ_α and/or λ_β.

Computation of seperatrices and switch-planes

The separatrices shown in Supplementary Fig. 26 divide the $([\alpha], [\beta)$-plane in two regions; Solutions of the reduced model (with $[\gamma] = [\delta] = 0$ nM) starting in the region below a seperatrix converge to the steady-state with positive $[\alpha]$ and zero $[\beta]$, whereas solutions of the reduced model (with $[\gamma] = [\delta] = 0$ nM) with initial conditions in the region above a seperatrix converge to the steady-state with zero $[\alpha]$ and positive $[\beta]$. The seperatrices are computed using the following procedure:

1. Determine the positive steady-state solution (which is a saddle-point) and denote this positive steady-state by x_{ss};
2. Determine the Jacobian matrix at x_{ss} and compute the eigenvalues and eigenvectors;
3. Let u be the eigenvector corresponding to the negative eigenvalue of the Jacobian and normalize u such that $\|u\| = 1$, then integrate the differential equations in negative t direction with initial conditions $x_{ss} + 10^{-8}u$ and $x_{ss} - 10^{-8}u$[1].

Whether or not a switch from $[\alpha]$ to $[\beta]$ or vice versa occurs can not be directly predicted from the location of the seperatrix; a switch is initiated by (the initial amplitude of) $[\gamma]$ or $[\delta]$ rather than by controlling the initial conditions in the $([\alpha],[\beta])$-plane (Supplementary Fig. 28).

To gain further insights in whether a switch will be successful or not we compute the so-called *switch-planes*. These switch-planes are global invariant manifolds in the $([\gamma], [\alpha], [\beta])$-space or $([\delta], [\alpha], [\beta])$-space for switching from β to α or from α to β respectively. As the switch-planes are global invariant manifolds, they divide the corresponding space in two parts; The part below the switch-planes defines the points at which switches can not occur whereas for points above the switch-planes there will be a switch (Supplementary Fig. 29). Note that the intersection of the switch-planes with the $([\alpha],[\beta])$-plane should be the seperatrix (that we have computed before).

We shall briefly discuss the computation of the $[\gamma]$-switch-plane. (Computation of the $[\delta]$-switch-plane is done analogously.) In other words, we consider only switches from β to α such that we can set $[\delta] \equiv 0$ (and, consequently, $\frac{d}{dt}[\delta] \equiv 0$). Thus the dynamics are described by the three-dimensional system of ODEs:

$$
\begin{aligned}
\tfrac{d}{dt}[\gamma] &= -\frac{V_{exo}[\gamma]}{K_{exo} + [\gamma] + [\alpha] + f_{i\alpha}([\alpha],[\beta]) + f_{i\beta}([\alpha],[\beta]) + f_\sigma([\alpha],[\beta])}, \\
\tfrac{d}{dt}[\alpha] &= \frac{V_\gamma[\gamma]}{K_\gamma + [\gamma]} + \frac{V_\alpha[\alpha]}{K_\alpha + [\alpha] + \lambda_\alpha f_{i\alpha}([\alpha],[\beta])} - (1-\rho)\frac{V_{L_\alpha}[\alpha]}{K_{L_\alpha} + [\alpha]} \\
&\quad - \frac{V_{exo}[\alpha]}{K_{exo} + [\gamma] + [\alpha] + [\beta] + f_{i\alpha}([\alpha],[\beta]) + f_{i\beta}([\alpha],[\beta]) + f_\sigma([\alpha],[\beta])}, \\
\tfrac{d}{dt}[\beta] &= \frac{V_\beta[\beta]}{K_\beta + [\beta] + \lambda_\beta f_{i\beta}([\alpha],[\beta])} - (1-\rho)\frac{V_{L_\beta}[\beta]}{K_{L_\beta} + [\beta]} \\
&\quad - \frac{V_{exo}[\beta]}{K_{exo} + [\gamma] + [\alpha] + [\beta] + f_{i\alpha}([\alpha],[\beta]) + f_{i\beta}([\alpha],[\beta]) + f_\sigma([\alpha],[\beta])}.
\end{aligned} \tag{18}
$$

Because the switch-plane is the union of trajectories that converge to points on the seperatrix we can approximate the switch-planes by

[1]Let $\phi(t;x)$ be a solution of the systems through x. The stable manifold of x_{ss} is the set $\mathcal{W}_s(x_{ss}) := \{x : \phi(t;x) \to x_{ss} \text{ as } t \to \infty\}$, the unstable manifold of x_{ss} is the set $\mathcal{W}_u(x_{ss}) := \{x : \phi(t;x) \to x_{ss} \text{ as } t \to -\infty\}$. As the positive steady-state is a saddle-point, invoking the *stable and unstable manifold theorem*,[6] $\mathcal{W}_s(x_{ss})$ and $\mathcal{W}_u(x_{ss})$ are tangent to the stable, respectively, unstable eigenspaces of the linearization at x_{ss}.

1. taking initial conditions for $[\alpha]$ and $[\beta]$ on the seperatrix and set the initial condition for $[\gamma]$ to be 10^{-8};
2. integrate the three-dimensional system of ODEs in negative t direction.

It is interesting to note that the effect of asymmetry in the model (as the parameters of the α-side of the switch are not identical to those of the β-side) can be compensated for by coupling the translator to the β-side (Supplementary Figure 29d-e). Furthermore, in case of the translator being coupled to the α-side a (unrealistically) high amplitude is needed for successful switching from β to α (Supplementary Fig. 29b) and, in particular, Supplementary Fig. 29c).

INVERTER

From a modelling perspective the INVERTER circuit can be considered as a special case of the switch with the translator coupled to the α-side. Indeed, setting parameter $V_\beta = 0$ (removing the autocatalytic loop at β) and letting the initial amplitudes of $[\gamma]$ and $[\delta]$ being zero (which implies that both $[\gamma]$ and $[\beta]$ remain zero) we arrive at the following heuristic dynamic model of the INVERTER circuit:

$$
\begin{aligned}
\tfrac{d}{dt}[\alpha] &= \frac{V_\alpha[\alpha]}{K_\alpha + [\alpha] + \lambda_\alpha[i\alpha]} - \frac{V_{exo}[\alpha]}{K_{exo} + [\alpha] + [\beta] + [i\alpha] + [i\beta] + [\sigma]} - \frac{V_{L_\alpha}[\alpha]}{K_{L_\alpha} + [\alpha]} + k_\alpha[\theta],\\
\tfrac{d}{dt}[\beta] &= -\frac{V_{exo}[\beta]}{K_{exo} + [\alpha] + [\beta] + [i\alpha] + [i\beta] + [\sigma]},\\
\tfrac{d}{dt}[i\alpha] &= \frac{V_{i\alpha}[\beta]}{K_{i\alpha} + [\beta]} - \frac{V_{exo}[i\alpha]}{K_{exo} + [\alpha] + [\beta] + [i\alpha] + [i\beta] + [\sigma]},\\
\tfrac{d}{dt}[i\beta] &= \frac{V_{i\beta}[\alpha]}{K_{i\beta} + [\alpha]} - \frac{V_{exo}[i\beta]}{K_{exo} + [\alpha] + [\beta] + [i\alpha] + [i\beta] + [\sigma]},\\
\tfrac{d}{dt}[\sigma] &= \frac{V_{L_\alpha}[\alpha]}{K_{L_\alpha} + [\alpha]} - \frac{V_{exo}[\sigma]}{K_{exo} + [\alpha] + [\beta] + [i\alpha] + [i\beta] + [\sigma]} - k_\sigma[\sigma][MB],\\
\tfrac{d}{dt}[\theta] &= \rho\frac{V_{L_\alpha}[\alpha]}{K_{L_\alpha} + [\alpha]} - k_\alpha[\theta],\\
\tfrac{d}{dt}[MB] &= -k_{rep}[\sigma][MB],\\
\tfrac{d}{dt}[\sigma MB] &= k_{rep}[\sigma][MB].
\end{aligned}
\tag{19}
$$

As shown in Figure 3, this heuristic model describes the dynamics of the INVERTER circuit qualitatively well with the same parameters as used for the switch except that the value of ρ is increased (less inherent retroactivity). This can be explained by the competition between polymerase and nickase for their substrates for which the exact mechanism is unknown. However, it is known the activity of the enzymes are affected by competition, e.g. nickase can bind to the substrate of polymerase and, therefore, a higher nickase concentration not necessarily results in faster kinetics (Supplementary Fig. 30). Moreover, this effect is also dependent on the template concentration (Supplementary Fig. 30). Hence, it is likely the kinetics of polymerase and nickase, which determine ρ, vary in different systems.

Supplementary References

1. Padirac, A., Fujii, T. & Rondelez, Y. Bottom-up construction of in vitro switchable memories. *Proc. Natl. Acad. Sci. U.S.A.* **109**, 3212-3220 (2012).

2. Engelen, W., van de Wiel, K.M., Meijer, L.H.H., Saha, B. & Merkx, M. Nucleic acid detection using BRET-beacons based on bioluminescent protein-DNA hybrids. *Chem. Commun.* **53**, 2862-2865 (2017)

3. Janssen, B.M.G., Engelen, W. & Merkx, M. DNA-directed control of enzyme–inhibitor complex formation: a modular approach to reversibly switch enzyme activity. *ACS Synth. Biol.* **4**, 547-553 (2015).

4. Chen, Y.J. & Huang, X. DNA sequencing by denaturation: principle and thermodynamic simulations. *Anal. Biochem.* **384**, 170-179 (2009).

5. Zhang, D.Y. & Winfree, E. Control of DNA strand displacement kinetics using toehold exchange. *J. Am. Chem. Soc.* **131**, 17303-17314 (2009).

6. Guckenheimer, J. & Holmes, P.J. *Nonlinear Oscillations, Dynamical Systems, and Bifurcations of Vector Fields*, (Springer-Verlag New York, 2002).
